# CeO_2_‐Based Electrocatalysts for the Oxygen Evolution Reaction: Design, Dynamic Mechanisms, and Electrolyte Adaptability

**DOI:** 10.1002/advs.78316

**Published:** 2026-10-18

**Authors:** Zhen Cui, Ailing Song, Zirui Cui, Manman Duanmu, Zhipeng Ma, Hao Liu, Guangjie Shao, Xinyi Tan, Guoxiu Wang, Hao Tian

**Affiliations:** ^1^ Hebei Key Laboratory of Applied Chemistry College of Environmental and Chemical Engineering Yanshan University Qinhuangdao China; ^2^ State Key Laboratory of Metastable Materials Science and Technology Yanshan University Qinhuangdao China; ^3^ Centre For Clean Energy Technology School of Mathematical and Physical Sciences Faculty of Science University of Technology Sydney Broadway NSW Australia

**Keywords:** CeO_2_‐based electrocatalysts, interface engineering, lattice evolution, oxygen evolution reaction, surface reconstruction

## Abstract

CeO_2_‐based electrocatalysts are promising for the oxygen evolution reaction (OER) owing to their distinctive Ce^3+^/Ce^4+^ redox flexibility, large oxygen‐storage capacity, and lattice adaptability. More importantly, CeO_2_ can dynamically regulate catalyst surface reconstruction and electronic structure while significantly enhancing catalytic activity and long‐term stability. In this review, we first focus on CeO_2_‐based electrocatalysts for OER from structural characteristics, design principles and strategies, structure–property relationships, interfacial structure, as well as critical evaluation of design strategies and selection principles. The mechanistic roles of CeO_2_ in OER are highlighted, including redox chemistry and oxygen‐vacancy (O_v_) dynamics, electronic‐structure modulation and charge transfer, interfacial coupling and surface reconstruction, as well as operando evidence and the controversy over lattice‐oxygen participation. The performance of CeO**_2_**‐based OER electrocatalysts in different electrolytes, including alkaline, acidic, and seawater media, is also summarized to demonstrate their applicability under diverse operating conditions. Finally, from the perspectives of electronic‐structure evolution, redox‐state transitions, lattice evolution, and catalytic pathways, this review summarizes the underlying mechanisms by which CeO_2_ regulates the dynamic catalytic behavior of OER and provides an outlook on future design strategies for developing highly efficient and durable CeO_2_‐based electrocatalysts.

## Graphical abstract

This Review shows how cerium oxide helps water‐splitting catalysts work better and last longer. It explains how CeO_2_ tunes electronic structure, oxygen‐vacancy behavior, and surface reconstruction, while adapting to alkaline, acidic, and seawater conditions. By linking structural design with dynamic catalytic mechanisms, the Review outlines strategies for developing more efficient and durable oxygen‐evolution electrocatalysts.

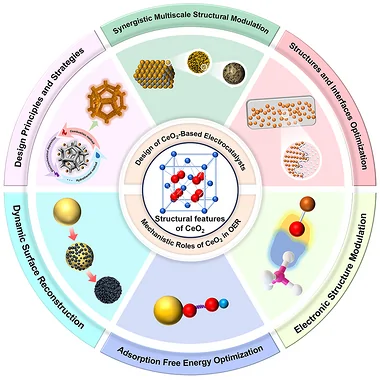

## 1 Introduction

In renewable energy systems such as water electrolyzers, fuel cells, and metal–air batteries, the oxygen evolution reaction (OER) stands as a half‐reaction of pivotal significance [1, 2, 3, 4, 5]. Alkaline water electrolysis, specifically, has drawn intense interest as a clean and enduring pathway for large‐scale hydrogen production driven by renewable power. This development is widely regarded as a linchpin of the emerging hydrogen economy [6, 7, 8]. Yet translating this promise into practice is fundamentally constrained because OER suffers from sluggish kinetics and high overpotential, thereby imposing a stubborn efficiency ceiling. As shown in Figure 1a, at its root, the OER, as a four‐electron transfer process (4OH^−^ → O_2_ + 2H_2_O + 4e^−^), derives its slow kinetics from multi‐scale complexity [9, 10, 11, 12, 13]. More specifically, the reaction typically proceeds through a series of intermediates such as ^*^OH, ^*^O, and ^*^OOH. It encompasses a multitude of factors intricately intertwined, including the O‐H bond activation, the electron‐transfer kinetics of each elementary step, the adsorption affinities of the key intermediates, and the extent of proton‐electron transfer. Together, these tightly coupled contributions give rise to kinetic barriers that stretch across both temporal and spatial scales [12, 13, 14]. Within this context, it proves exceptionally difficult for an individual catalytic system to, all at once, refine the adsorption energetics of assorted intermediates and maintain structural robustness under highly oxidizing potentials. This fundamental hurdle imposes stringent limits on both catalytic activity and durability during practical use. Cocatalysts, however, can effectively coordinate these multi‐scale processes: they not only optimize the local electronic structure, but also modulate the synergistic efficiency of energy transfer pathways at different hierarchical levels, ultimately breaking through the intrinsic bottlenecks that limit the OER rate [15, 16, 17]. Therefore, it is imperative to introduce cocatalysts through modification strategies to construct multi‐component synergistic interactions, thereby enhancing OER performance [18, 19, 20, 21].

**FIGURE 1:**
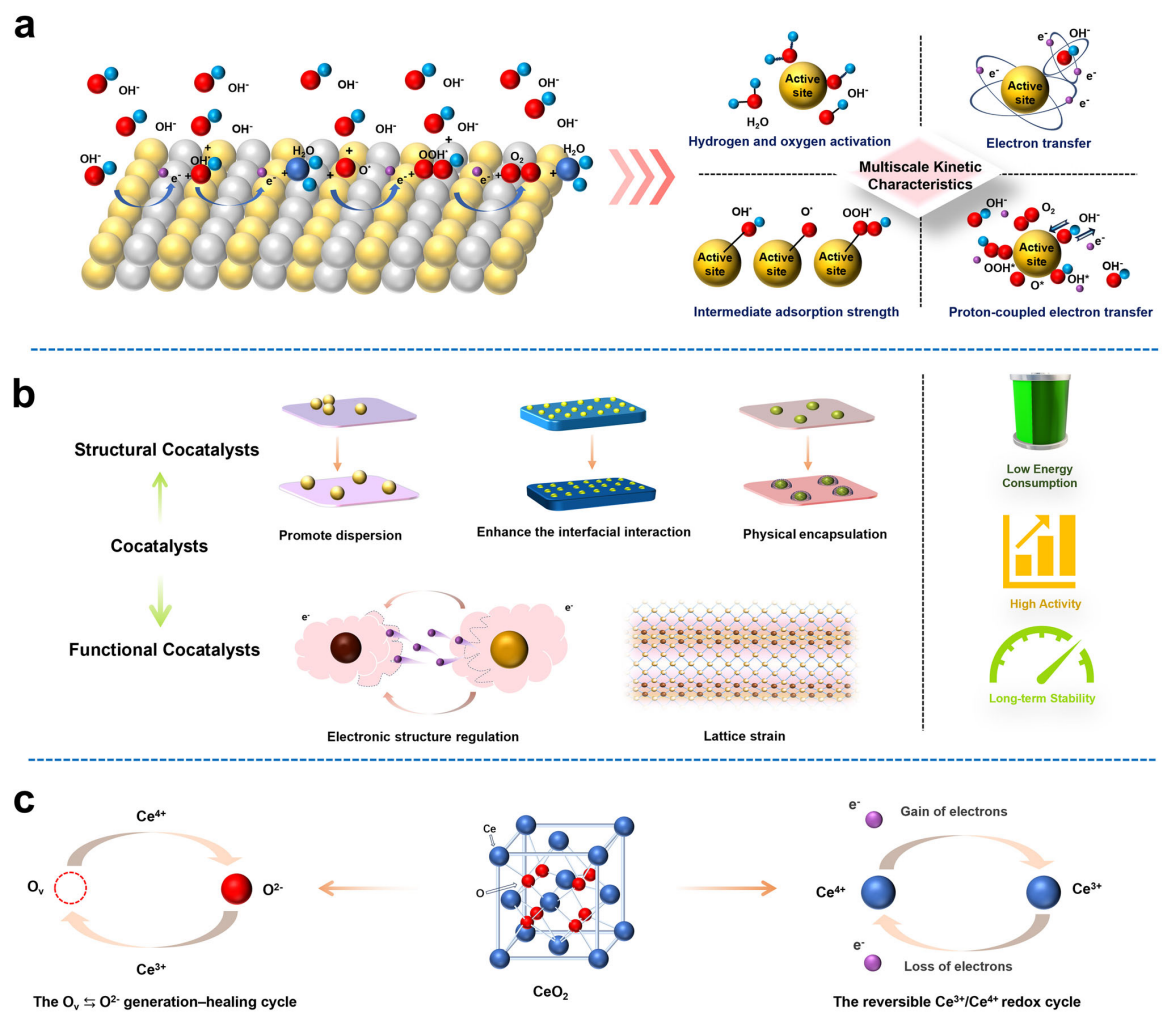
(a) The OER process of catalysts in alkaline solution and the multiscale kinetic characteristics of the OER, including hydrogen and oxygen activation, electron transfer, intermediate adsorption strength and proton‐coupled electron transfer. (b) Classification of cocatalysts into structural cocatalysts (promoting dispersion, enhancing interfacial interaction, physical encapsulation) and functional cocatalysts (electronic structure regulation, lattice strain), and the resulting benefits. (c) The O_v_ / O^2−^ generation‐healing cycle and the reversible Ce^3+^/Ce^4+^ redox cycle of CeO_2_.

Introducing cocatalysts into catalyst systems has emerged as a highly effective modification strategy [22, 23, 24, 25, 26, 27, 28]. As shown in Figure 1b, based on their mechanistic roles, cocatalysts can be categorized into structural cocatalysts (e.g., hexagonal boron nitride stabilizing active component dispersion through physical encapsulation [29], Ir‐Ta‐based metallic glass in situ constructing bilayer nanostructures, locking active‐site coordination environment [30]) and functional cocatalysts (e.g., Sr/Cr optimizing the electronic structure of active sites through bidirectional electronic regulation [31]). The core value of cocatalysts in the OER lies in coordinating the multi‐scale kinetic processes spanning from electron transfer to macroscopic transport [15, 16, 17]. On the one hand, interactions with the primary catalyst induce band bending and charge redistribution, significantly reducing interfacial charge transfer impedance (especially under high current density conditions) and ensuring efficient electron transport capability [32, 33, 34, 35, 36, 37, 38]. On the other hand, multiphase synergy allows one to fine‑tune the adsorption energy barriers of key oxygen‑containing intermediates (^*^OH, ^*^O, ^*^OOH). It might also reveal alternative reaction pathways, such as cation‑coupled electron transfer, which can bypass the limitations set by traditional scaling relationships [39, 40, 41, 42, 43, 44]. Furthermore, cocatalysts can serve as protective buffer layers, guarding against structural disintegration and metal leaching over extended electrolysis [45, 46, 47, 48, 49, 50, 51]. Nevertheless, such conventional interfacial regulation is largely established on a static structural and functional electronic framework, which may be insufficient to capture the complex evolution of catalytic interfaces under OER conditions. Cerium dioxide (CeO_2_), among the candidate materials, has become a representative rare‐earth promoter that serves structural roles owing to the high crustal abundance of Ce and its distinctive 4f‐orbital electronic configuration. Particularly, its unusual capacity under operando catalysis distinguishes it from traditional cocatalysts [52, 53, 54, 55, 56]. Its principal mechanistic contributions can be broken down into three areas: (1) Redox‐mediated dynamic cycling of O_v_. The O_v_ ⇆ O^2−^ generation–healing cycle, driven by the reversible Ce^3+^/Ce^4+^ redox couple of CeO_2_ (Figure 1c), not only enables rapid interfacial electron storage and release. By dynamically tuning lattice oxygen activity, this cycle also directly drives down the formation potential of the OER rate‐determining step [57, 58, 59]. (2) Synergistic regulation of electronics and adsorption at heterointerfaces: The oxophilic interface provided by CeO_2_ promotes the directional migration and enrichment of OH^−^ species, while the strong electronic coupling effect established with the primary catalyst optimizes the d‐band center position of active sites, thereby balancing intermediate adsorption energies and accelerating reaction kinetics [60, 61, 62, 63]. (3) Self‐healing stability based on the fluorite structure: Its stable calcium fluoride crystal structure imparts excellent corrosion resistance, and the self‐healing mechanism of O_v_ effectively compensates for surface oxygen loss and anchors metal single atoms/nanoclusters, thereby suppressing metal leaching and irreversible surface reconstruction under harsh alkaline conditions and preserving structural integrity over long‐term service [54, 55, 56, 57, 58, 59, 60]. It is precisely this coupled regulation of electronic structure, redox and lattice oxygen chemistry, and self‐healing properties that distinguishes CeO_2_ from conventional static cocatalysts and makes it a promising platform for highly efficient and cost‐effective CeO_2_‐based OER electrocatalysts [64, 65, 66].

To gain an in‐depth understanding of the promotional mechanism of CeO_2_ in the OER, this review systematically surveys the design principles and strategies, interfacial structures, structure–activity relationships, as well as critical evaluation of design strategies and selection principles of CeO_2_‐based OER catalytic systems organized by their structural features. Existing reviews have mainly provided systematic summaries of Ce‐based catalysts in terms of material classification, synthesis methods, morphological control, defect and oxygen vacancy engineering, heterostructure construction, as well as the effects of Ce doping, supports, and supported structures on OER activity and stability [67, 68, 69, 70, 71, 72, 73, 74]. Diverging from these reviews, which primarily focus on “how materials are constructed and how structural regulation enhances performance,” this review further highlights the dynamic evolution of CeO_2_‐based OER catalytic systems from their initial structures to their real working states and links this dynamic evolution to functional transitions under different electrolyte environments. Specifically, this review integrates CeO_2_ in different forms including as a support, a heterogeneous component, a doped phase, or a main component into a unified analysis. Centered on the reversible Ce^3+^/Ce^4+^ redox cycle and the dynamic evolution of O_v_, and combined with the lattice oxygen chemistry of the host catalyst, it constructs a dynamic analytical framework of the “redox–lattice oxygen interaction,” further correlating electronic structure modulation, interfacial charge transfer, oxygen vacancy generation and migration, and adaptive surface reconstruction, and revealing how CeO_2_ progressively transforms from a functional component in the initial structure into a dynamic regulatory unit that governs active centers and interfacial structural evolution under OER working conditions. Subsequently, this review systematically discusses O_v_ generation, electronic structure modulation, surface reconstruction, as well as operando evidence and the controversy over lattice‐oxygen participation, and further compares the performance of CeO_2_‐based OER electrocatalysts in alkaline, acidic, and seawater electrolytes and their environment‐dependent roles. Finally, it identifies the core challenges that remain and outlines future research directions. By integrating structural design, dynamic evolution under working conditions, and environmental adaptability, this review aims to provide systematic mechanistic understanding and design principles for understanding the structure–activity relationships of CeO_2_‐based OER electrocatalysts under practical reaction conditions and for developing catalytic systems that combine high activity, long‐term stability, and adaptability to complex environments.

## 2 Structural Characteristics and Design of CeO_2_‐Based Electrocatalysts

As shown in Figure 2, the performance of CeO_2_‐based OER electrocatalysts is ultimately determined by their structural composition and the underlying design principles that modulate catalytic activity across multiple scales, from the atomic to the macroscopic. Rational structural design aims to optimize active‐site accessibility, electronic structure, and mass‐transport pathways. This is achieved through deliberate modulation of morphology, surface defects, and interfacial architecture. CeO_2_‐based electrocatalysts can be structured as nanosheets, porous frameworks, and other morphologies that provide high surface areas and well‐defined, stable interfaces. These features are crucial for maximizing the exposure of catalytic sites and facilitating charge transfer at interfaces. The structural advantages are vital for enhancing the accessibility of active sites, promoting electronic transport, and accelerating reaction kinetics. However, designing highly efficient catalysts under demanding electrochemical conditions requires balancing intrinsic catalytic activity with structural stability, representing a complex multiscale optimization challenge. At the macro‐scale, hierarchical porosity optimizes mass transport of reactants and products; at the meso‐scale, nanostructures such as nanowires and nanosheets expose high‐activity facets to the greatest extent; at the atomic level, doping heteroatoms or anchoring single atoms allows precise tuning of electronic structures and local coordination environments. Beyond these intrinsic factors, the engineering of composite structures and interfaces is equally critical to overall catalytic performance. When CeO_2_ is integrated with other functional materials via heterostructures, core‐shell architectures, or solid solutions, its abundant O_v_s and reversible Ce^3+^/Ce^4+^ redox properties confer electron‐buffering capacities that can modulate interfacial charge transfer and reaction energetics. Collectively, these insights establish a coherent framework linking structural features to catalytic function, laying a foundation for subsequent discussions on the structural characteristics, design principles, and interfacial phenomena of CeO_2_‐based OER electrocatalysts.

**FIGURE 2:**
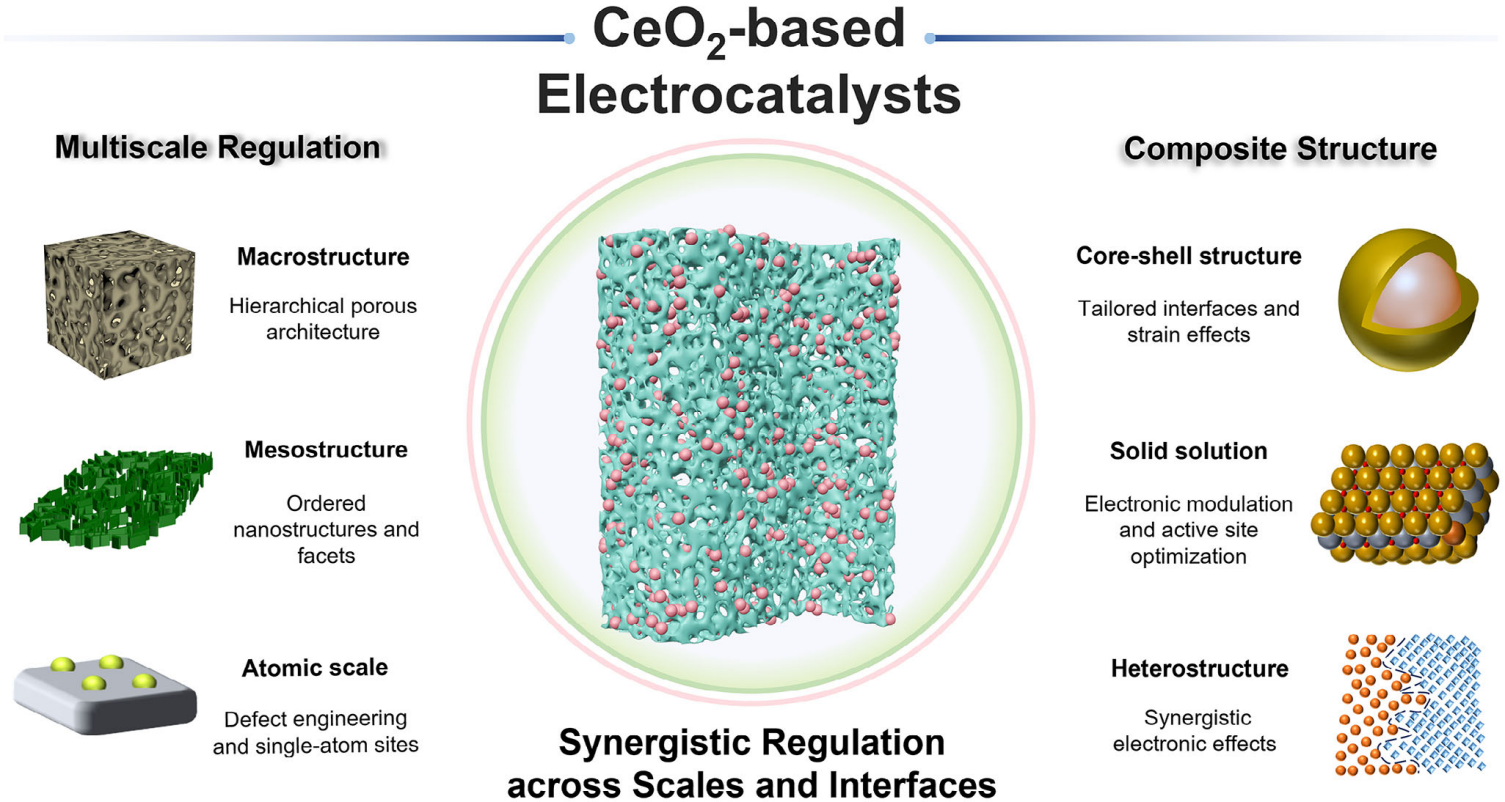
The structural design and synthesis strategies of CeO_2_‐based electrocatalysts for OER.

### 2.1 Structural Features

As shown in Figure 3a, CeO_2_ adopts the fluorite structure (space group Fm‐3m), in which every Ce^4+^ is coordinated by eight O^2−^ anions, forming an open‐framework architecture. This configuration not only endows the material with significant thermal and chemical stability but also facilitates reversible oxygen absorption and desorption via Ce^3+^/Ce^4+^ redox cycling, complemented by a high density of intrinsic O_v_, and local coordination adjustment, enabling CeO_2_ to achieve lattice adaptability while maintaining the stability of the fluorite structure [67, 68, 69, 70, 71, 72, 73, 74, 75, 76]. Looking at the electronic‐structure origins, the Ce 4f orbitals are strongly localized, and their energy level sits right between the O 2p valence‐band maximum and the transition‐metal 3d orbitals. This energetic positioning triggers a cascade: it first creates a with a low calculated barrier Ce^3+^/Ce^4+^ couple and, via O 2p bridging, establishes a gradient “d‐p‐f” electronic coupling conduit to the transition metal (TM) 3d states (Figure 3b). Taking the TM/CeO_2_ heterojunction as an example, the TM 3d and Ce 4f orbitals each couple with O 2p via σ‐ and π‐type bonding, which establishes a 3d–4f gradient orbital linkage right across the interface (Figure 3c). The favorable energetic alignment enables the Ce 4f states to act as an electron buffer, thereby facilitating d–p–f orbital coupling and providing an effective route for precise modulation of the interfacial electronic structure [77, 78, 79, 80, 81, 82]. Under OER polarization, Ce^4+^ (4f^0^) can capture electrons rapidly and be reduced to Ce^3+^ (4f^1^), and the reverse transformation proceeds with comparable efficiency. The redox pair acts as an electronic buffer valve: it soaks up excess charge accumulating on the TM sites of the host catalyst, thereby curbing peroxidation, while simultaneously releasing electrons in the reverse direction to sustain high‑valent active intermediates [83, 84]. Exploiting the compositional tunability of the fluorite lattice, the incorporation of lower‐valent or size‐mismatched transition‐metal cations into the CeO_2_ matrix induces marked lattice distortion and local strain [65]. Furthermore, lattice mismatch between the fluorite host and secondary phases often produces atomically abrupt heterointerfaces; these interfacial regions serve as privileged sites for oxygen‑vacancy enrichment and, through interfacial reconstruction, can generate unusual coordination environments or amorphous/crystalline dual‑phase motifs [85, 86, 87, 88, 89]. Owing to their facet‑selective growth habit, CeO_2_ fluorite crystals can self‐assemble into hierarchical architectures via hydrothermal or solvothermal routes, thereby dramatically boosting the specific surface area and the exposure of active sites. By optimizing synthetic strategies, CeO_2_‐based electrocatalysts with tailored nanostructures can be rationally fabricated [65, 66, 67, 68, 69, 70, 71, 72, 73, 74, 75, 76, 77, 78, 79, 80, 81, 82, 83, 84, 85, 86, 87, 88, 89, 90]. It should be stressed that this fluorite structure displays pronounced corrosion resistance in alkaline seawater environments. When deployed as a protective shell, CeO_2_ effectively shields the catalytically active core from poisoning by Cl^−^ and other impurities, thereby ensuring high durability during seawater electrolysis [86].

**FIGURE 3:**
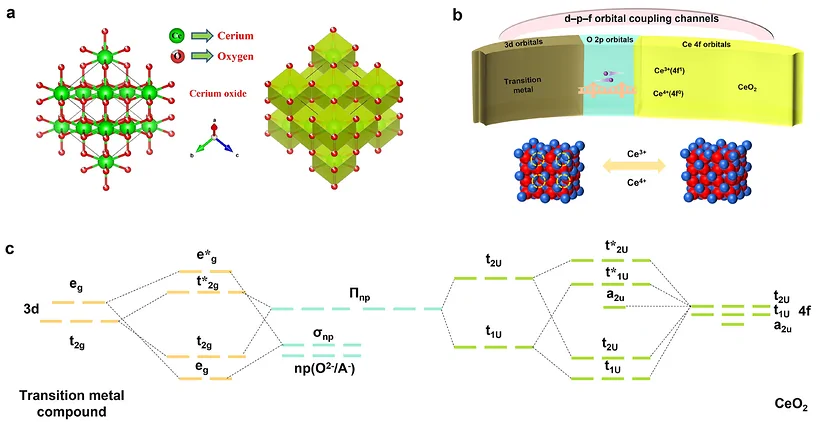
(a) Crystal structure of CeO_2_ in fluorite‐type cubic arrangement (space group Fm‐3m) at room temperature and ambient conditions. Reproduced with permission [76]. Copyright 2024, Springer Nature. (b) Simplified schematic diagram of orbital coupling between transition metal 3d and CeO_2_. (c) Schematic diagram of the gradient orbital coupling mechanism between transition metal 3d and ceria 4f. Reproduced with permission [77]. Copyright 2024, The Royal Society of Chemistry.

### 2.2 Design Principles and Strategies

Optimizing the OER performance of CeO_2_‐based electrocatalysts hinges upon meticulous structural engineering, guided by well‐chosen synthesis strategies. At the core of the design is a drive to establish clear, causal links between synthesis conditions and microstructural traits, so that high activity, long‐term stability, and resilience across aggressive environments can be achieved all at once, particularly strongly acidic or alkaline electrolytes [62, 91, 92]. Structural manipulation mainly targets three aspects: the interfacial contact between CeO_2_ and active components, the geometric architecture, and the density of O_v_; together, these determine both the inherent activity of catalytic centers and their durability under operation [72, 93, 94]. Picking a synthetic method thus boils down to striking a balance among active‐site accessibility, interfacial coupling strength, and the structural robustness demanded by prolonged service. A few proven synthetic pathways have emerged that permit fine control over composition‐structure‐property relationships. Hydrothermal and solvothermal approaches are a mainstay for constructing CeO_2_‐based heterostructures, which are favored for their experimental simplicity. Here, the underlying logic is to tune thermodynamic and kinetic handles, such as precursor concentration, reaction temperature and dwell time, in order to steer the formation of well‑defined morphologies (e.g., nanosheets, nanospheres) and sharp heterointerfaces [85, 95, 96]. As shown in Figure 4a, electrodeposition works by a different mechanism: it relies on in situ nucleation and crystallization directly on a conductive substrate, which markedly tightens interfacial electron‐transport channels. This in situ nature makes the technique particularly attractive for the integrated design of self‑supporting electrodes [97]. The molten salt method, for its part, draws on the high ionic conductivity and fluidity of a molten salt bath to accelerate elemental mass transport and crystal growth. Under high‑temperature chemical conditions, such an environment permits deep‐seated adjustments of crystal structure and micromorphology that, in turn, boost catalytic performance [98]. When targeting real‐world electrolysis, structural design must also contend with electrolyte corrosion. In strongly alkaline environments, the emphasis of structural engineering pivots to utilizing defect engineering to modulate the local electronic structure of CeO_2_. Such modulation effectively suppresses structural collapse caused by the oxidation of Ce^3+^, thereby sustaining long‐term operational stability at elevated current densities [59]. Conversely, in strongly acidic media, severe corrosion and dissolution pose critical challenges, while structural tailoring relies on lattice matching strategies and strain engineering. By respectively adjusting the lattice parameters and interfacial stress between CeO_2_ and metal oxide counterparts, these approaches synergistically enhance both the tolerance and activity of the catalyst under acidic oxygen evolution potentials [99, 100]. Notably, to overcome the intrinsic limitations of conventional wet‐chemical methods in crystallinity control and interfacial coupling strength, several innovative auxiliary synthetic techniques have begun to intervene in structural regulation at increasingly microscopic scales. For instance, the transient high‐temperature and high‐pressure microenvironments generated by ultrasonic cavitation effects can induce the exposure of high‐energy, highly reactive crystal facets on the CeO_2_ support surface while simultaneously inducing surface roughening. When combined with low‐temperature electron tomography for near‐native‐state monitoring of the penetration depth of the primary catalyst within the CeO_2_ lattice, this approach enables atomically precise anchoring of active components onto the support surface [101]. Additionally, laser direct writing technology utilizes the localized thermal effect of millisecond pulsed laser irradiation to in situ induce the formation of hierarchical nanosheet arrays on CeO_2_ substrates, while simultaneously allowing for the precise regulation of O_v_ concentrations (Figure 4b) [102]. Although the aforementioned cutting‐edge strategies have yet to achieve full industrial maturity, these emerging methods establish a new paradigm for atomic‐scale structural manipulation, providing the requisite theoretical foundations and design concepts to circumvent the limitations of conventional synthesis.

**FIGURE 4:**
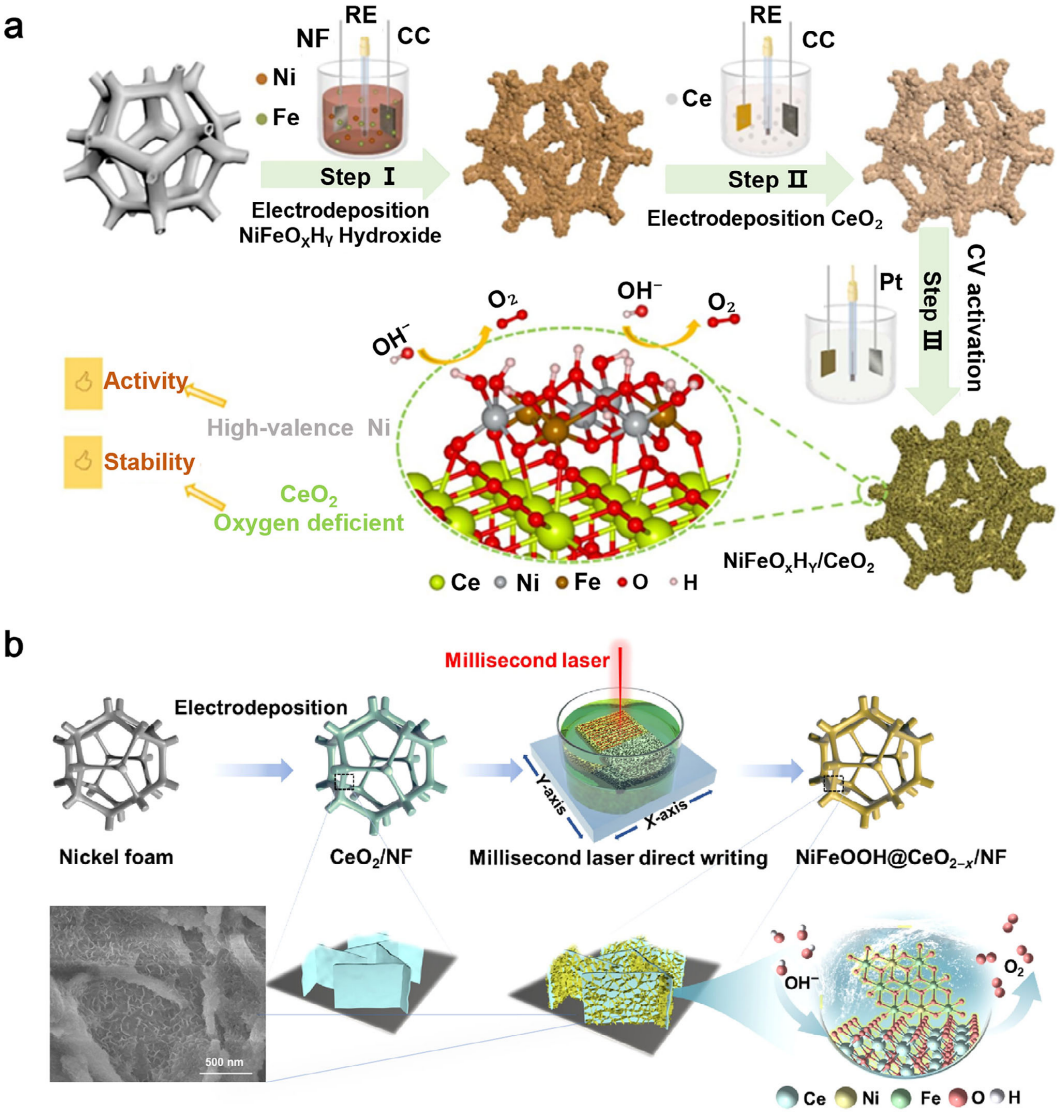
(a) Traditional synthesis methods of CeO_2_‐based materials, schematic diagram of the electrodeposition process for synthesizing NiFeO_x_H_y_/CeO_2_ OER catalysts. Reproduced with permission [97]. Copyright 2024, Elsevier. (b) Innovative synthesis methods of CeO_2_‐based materials. The synthetic route of hierarchical NiFeOOH@CeO_2‐x_ nanosheets arrays and SEM image of NiFeOOH@CeO_2‐x_/NF. Reproduced with permission [102]. Copyright 2025, Tsinghua University Press.

### 2.3 Structure‐Property Relationships

Optimizing catalyst structures across spatial dimensions is the central approach to boosting the OER activity of CeO_2_‐based materials, and it calls for a coordinated design that bridges macroscopic morphology down to atomic arrangements. At the macroscopic end, constructing hierarchical pore networks dramatically raises the fraction of accessible active sites while accelerating mass transport (Figure 5a–c) [103, 104]. This advantage depends on matching pore dimensions with connectivity: smaller pores provide accessible surfaces, connected larger pores facilitate electrolyte supply and oxygen removal, and the continuous solid framework supports electron transport. Bubble behavior should therefore be considered rather than pursuing high porosity alone [105]. Such architectures can be realized through dealloying and templating. In dealloying, selective etching together with high‐temperature phase transformations creates a three‑dimensionally interconnected pore system, markedly enhancing mass transfer [106]. Templating routes, for instance those derived from MOFs, use high‐temperature calcination to yield porous oxide composites that inherit the high specific surface area and porosity of the precursor [107]. Pore dimensions, pore throats, and wall thickness can be adjusted through template size and packing, the extent of etching, and calcination conditions. A load‐bearing framework and firm interfaces should be retained to reduce the risk of fracture and detachment caused by excessive pore formation. Studies of hierarchical porous Ni‐based OER electrodes demonstrate that surface pore formation can be compatible with mechanical integrity [108]; prolonged electrolysis using porous rare‐earth high‐entropy perovskites provides a complementary example [109]. Descending to the nanoscale through mesoscopic structural engineering brings precise morphological control. Configurations such as one‐dimensional nanowires, two‐dimensional nanosheets, and three‐dimensional nanoarrays or nanospheres enable directional tuning of active‑site distribution, exploit spatial confinement to optimize charge‑transport pathways, speed up reactant diffusion, and preferentially expose highly reactive crystal facets [53]. Hydrothermal and solvothermal synthesis provides fine command over morphology and heterostructures (e.g., nanosheets, nanospheres) simply by adjusting precursor concentration, reaction temperature, and time [85, 95, 96]. A representative case is CeO_2_‐coated CoSe_2_ nanoneedles: a solvothermal process builds core‐shellCeO_2_@CoSe_2_ nanoneedles directly on carbon cloth, with the needles self‐aligning vertically into three‐dimensional flower‐like arrays, thereby substantially enlarging the electrochemically active surface area [85]. A study on the organic electrooxidation of CeO_2_@CuFe‐LDH provides a reference for the synergistic design of mesopores and interfacial charge transport: CeO_2_@CuFe‐LDH possesses mesopores of approximately 4.6 nm, which favor active‐site accessibility, while its crystalline‐amorphous heterointerface exhibits lower charge‐transfer resistance. Although this study focuses mainly on organic electrooxidation, its strategy of synergistically improving site utilization and charge transport through mesopore construction and interfacial coupling can offer inspiration for the structural design of CeO_2_‐based OER catalysts [110]. At the atomic scale, the emphasis shifts to atomistic precision. As shown in Figure 5d–f, a hollow, peanut‐shaped nitrogen‐doped carbon support is first prepared by a templating method, after which CeO_2_ nanoparticles are grown on its surface; subsequent high‐temperature annealing then confines and immobilizes atomically dispersed Ni species. This architecture merges the high exposure intrinsic to atomically dispersed Ni, the excellent electronic conductivity and mass‐transport channels offered by the hollow carbon framework, and a robust CeO_2_‐Ni interfacial interaction that stabilizes the metal centers [111]. Moreover, coupling ultrasonic cavitation with cryogenic electron tomography permits near‐native‐state observation of the subsurface anchoring depth of active components inside CeO_2_, which makes it possible to achieve sub‐nanoscale integration between the support and the active phase (Figure 5g–k) [101]. Defect engineering provides a particularly powerful atomic‐scale lever for CeO_2_‐based electrocatalysts because it can magnify intrinsic material properties. Through doping (hydrothermal/solvothermal routes), reductive treatments (H_2_ annealing), or morphological tailoring (acid/alkali etching), one deliberately engineers synergistic oxygen vacancy O_v_‐Ce^3+^ entities within the CeO_2_ lattice, thereby elevating the catalytic performance [112]. A review of lanthanum‐based OER materials likewise emphasizes coordinated defect, interface, and stability engineering [113]. An appropriate pore architecture should be determined at the intended current density by jointly assessing transport impedance, bubble detachment, and framework integrity after operation. In summary, designing high‐performance CeO_2_‐based OER electrocatalysts rests on a multi‐scale cooperative strategy, weaving together macroscopic morphological regulation, nanoscale pore engineering, and atomic‐level dispersion to deliver fully optimized OER performance from the macroscopic scale down to the atomic level.

**FIGURE 5:**
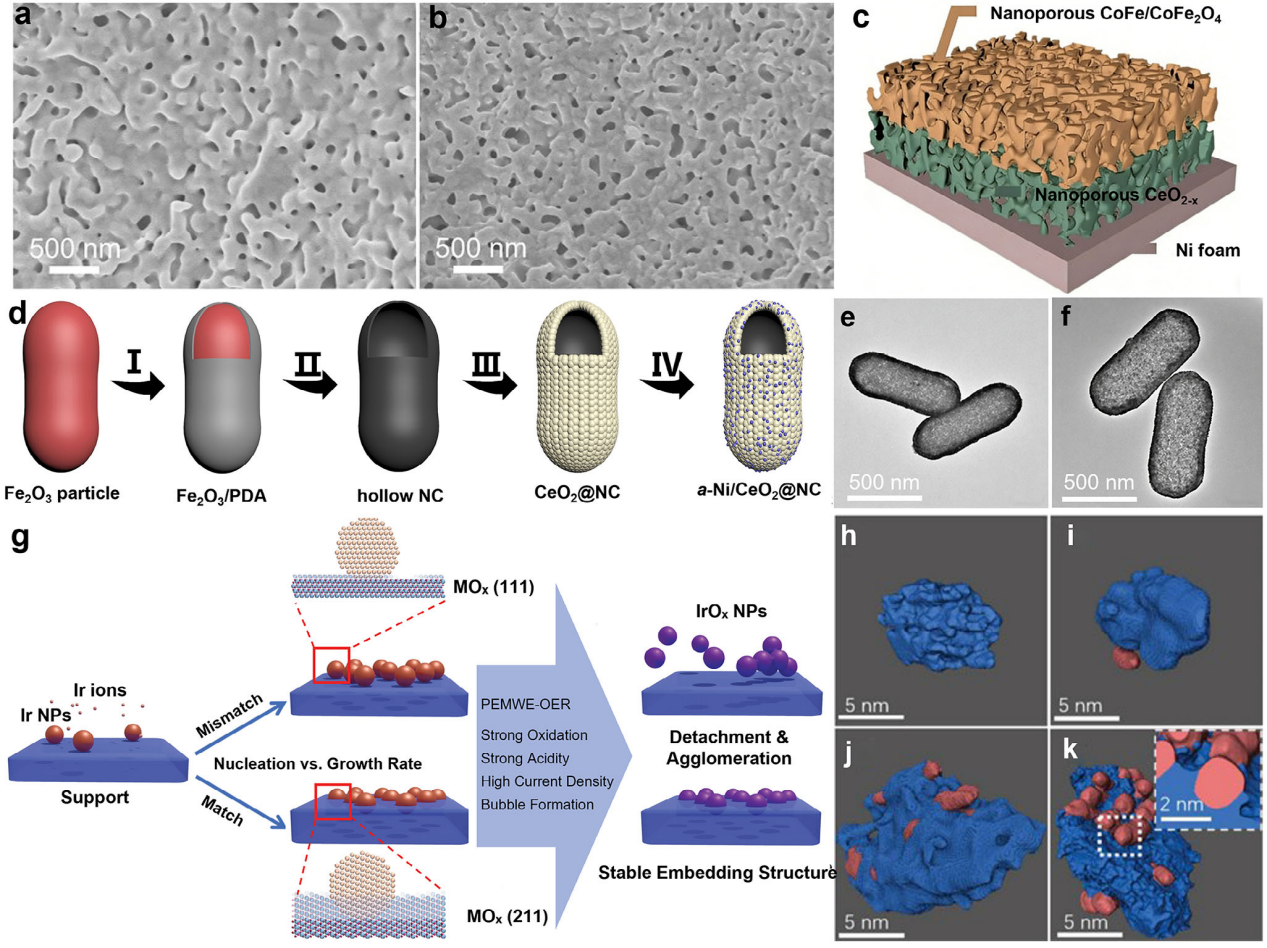
Multiscale structural engineering of CeO_2_‐based OER catalysts, from macroporous electrodes to atomically dispersed sites. (a–c) Macroscale: (a) SEM image of nanoporous CeO_2‐x_ prepared by self‐propagating solution combustion, showing the interconnected open pores that act as electrolyte reservoirs and gas‐release channels; (b) SEM image of the nanoporous CoFe/CoFe_2_O_4_ layer; (c) schematic of the layered heterostructured nanoporous electrode, illustrating how stacking the porous layers shortens ion‐diffusion paths while maintaining a continuous electron pathway. Reproduced with permission [103]. Copyright 2024, Springer Nature. (d–f) Atomic scale: (d) synthesis route of a‐Ni/CeO_2_@NC: (I) polydopamine (PDA) coating of peanut‐like Fe_2_O_3_ templates, (II) formation of hollow N‐doped carbon (NC) frameworks by annealing and HCl etching, (III) growth of CeO_2_ on NC, and (IV) atomic dispersion of Ni; (e, f) TEM images of (e) CeO_2_@NC and (f) a‐Ni/CeO_2_@NC, showing that the hollow NC framework provides conductivity and mass‐transport channels while CeO_2_ anchors atomically dispersed Ni through Ce‐O‐Ni interactions. Reproduced with permission [111]. Copyright 2023, AAAS. (g–k) Atomic deposition dynamics: (g) kinetic Monte Carlo (KMC) simulation of Ir nucleation with identical parameters on (111) and (211) metal‐oxide surfaces, rationalizing facet‐dependent anchoring; (h–j) cryo‐electron tomography (cryo‐ET) three‐dimensional reconstructions of the CeO_x_ support (blue) before and after 5 min and 60 min of Ir deposition (pink), that is, time‐resolved snapshots of samples frozen at defined deposition times, which reveal how the active phase progressively penetrates the support; (k) Cs‐corrected HAADF‐STEM image after 180 min of deposition (inset: atomic‐scale cross‐section). Reproduced with permission [101]. Copyright 2025, AAAS.

### 2.4 Interfacial Structure

CeO_2_ alone is not an efficient OER active phase. Combining it with synergistic components and constructing heterogeneous interfaces can fabricate highly catalytically active and stable OER electrocatalysts. This constitutes the fundamental approach to designing OER electrocatalysts, predicated on the redox characteristics of Ce, the kinetics of O_v_, and the adaptability of the lattice structure [37, 114]. As further extensions of the aforementioned properties, doping, solid‐solution formation, and heterojunction engineering are strategies for constructing such atomic and lattice‐matched/mismatched heterointerfaces. By creating atomic and lattice heterointerfaces, it is possible to enhance the CeO_2_‐like functionalities inherent to CeO_2_ itself or those inherited by replaced Ce within the lattices of other metal oxides. In practice, the large ionic radius of Ce within CeO_2_ together with the strict requirement for homogeneous and stable dopant incorporation renders Ce salt precursors the conventional doping route [115, 116, 117, 118]. As shown in Figure 6a, Ce‐doped transition metal oxides prepared through a hydrothermal process followed by calcination exhibit Ce substitution that preferentially targets the octahedral metal sites of spinel oxides (e.g., NiCo_2_O_4_ and Co_3_O_4_). This substitution induces lattice expansion and, importantly, introduces surface O_v_, driving charge redistribution. Such a structural arrangement permits precise manipulation of both the oxygen vacancy concentration and the metal valence states. Notably, it can even reroute the OER pathway from the conventional adsorbate evolution mechanism (AEM) to the kinetically more favorable lattice oxygen mechanism (LOM), bypassing the inherent scaling relationship limitation of AEM, ultimately bestowing exceptional OER activity and stability on the catalyst [119, 120]. Given the fluorite structure of CeO_2_, the construction of substitutional solid solution oxides likewise represents another common strategy for composite material design (Figure 6b). Using CeO_2_ as the host matrix, Ir atoms uniformly occupy Ce lattice sites to form a single‐phase structure, which, by virtue of the intrinsic structural stability of the solid solution and the characteristic properties of CeO_2_, simultaneously delivers high acidic OER activity and long‐term operational stability at low Ir loading, making it well‐suited for practical application in proton exchange membrane water electrolyzers [121]. Similar to these methods, heterointerface engineering plays a crucial role in the design of CeO_2_‐based electrocatalysts (Figure 6c–f). From a structural perspective, lattice matching/mismatching, interfacial strain, defect distribution, and geometric contact configurations in CeO_2_‐based heterointerfaces collectively govern O_v_ evolution, oxygen migration, active‐phase reconstruction, and interfacial stability [60, 99, 114, 122]. For example, Ru‐O‐Ce linkages and local lattice strain formed at the RuO_2_‐CeO_2_ interface can enhance interfacial structural stability, thereby favoring long‐term operation under acidic OER conditions [99]. Heterojunctions are routinely fabricated via hydrothermal, solvothermal, and electrodeposition techniques. By regulating the components, strains, defects, and geometric contact forms on both sides of the interface, the interfacial structure and oxygen kinetics behavior at the interface can be customized [111]. Therefore, the construction of CeO_2_‐based heterointerfaces can further regulate O_v_ evolution and active‐site reconstruction. CeO_2_ can be integrated with other components in several distinct configurations, for instance, as a supporting substrate or as a surface modifier [123, 124, 125, 126]. To illustrate the substrate role, one may consider a heterojunction formed between CeO_2_ and a transition metal oxide, such as CoO. Meanwhile, CeO_2_ acting as a surface modifier traps hydroxyl radicals through interfacial O_v_, accelerating O‐O bond formation [128]. Overall, rational construction of CeO_2_‐based interfacial structures can improve OER activity and structural stability by regulating lattice coupling, defect distribution, oxygen migration, and active‐phase reconstruction [60, 99, 111, 114, 122]. The charge transfer, built‐in electric fields, orbital coupling, and changes in adsorption energy that arise after interface formation are discussed collectively in Section 3.2 [114, 129]. Throughout this review, “lattice‐oxygen participation” refers to the lattice oxygen of the host catalyst unless the involvement of the own lattice oxygen of ceria is explicitly stated and supported by direct evidence.

**FIGURE 6:**
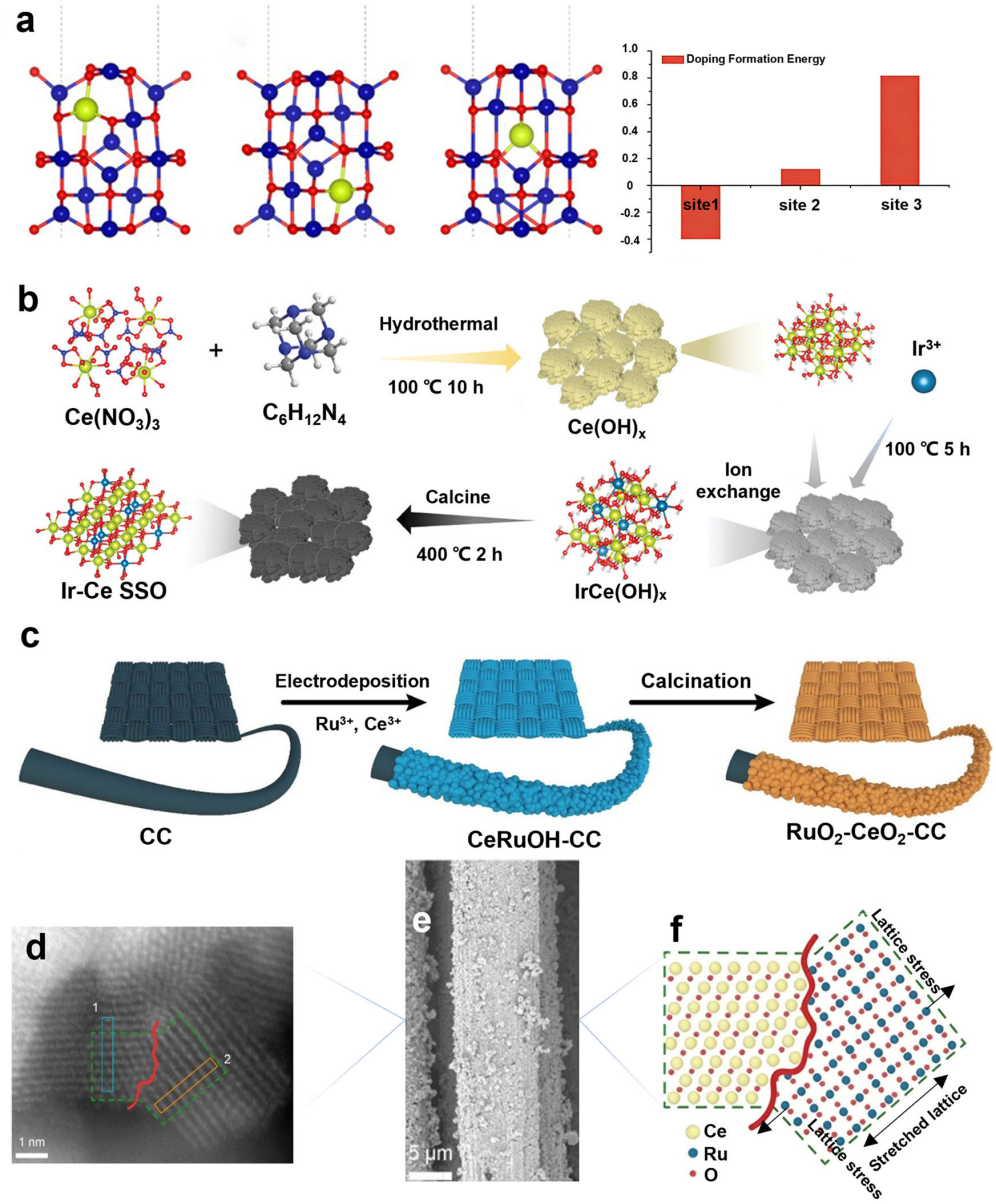
(a) Cerium doping strategy: Calculated formation energies for Ce doping at distinct sites in Co_3_O_4_. Reproduced with permission [120]. Copyright 2025, Elsevier. (b) Construction of Ce‐containing solid‐solution: Schematic synthesis route of Ir‐Ce oxide solid solution (Ir‐Ce SSO). Reproduced with permission [121]. Copyright 2024, John Wiley and Sons. (c–f) Heterostructure engineering of ceria‐based composites: (c) Schematic fabrication process of RuO_2_‐CeO_2_‐carbon cloth (CC) catalyst, (d) TEM image of RuO_2_‐CeO_2_‐CC, (e) SEM image of RuO_2_‐CeO_2_‐CC, (f) Atomic‐scale model of RuO_2_‐CeO_2_, corresponding to the green‐framed region in (d). Reproduced with permission [99]. Copyright 2024, American Chemical Society.

### 2.5 Critical Evaluation of Design Strategies and Selection Principles

The aforementioned Ce doping, O_v_ engineering, heterointerfaces, solid solutions, and morphology control can all improve the performance of CeO_2_‐based OER electrocatalysts, but their functional emphases and applicable conditions are not the same. Overall, Ce doping, moderate O_v_, and strongly coupled CeO_2_ heterointerfaces can directly regulate the local coordination of active centers, metal valence states, and interfacial electronic structure, and are therefore more favorable for enhancing intrinsic activity [78, 112, 119, 120, 127, 130, 131, 132]; hierarchical porous structures, self‐supported arrays, and morphology engineering mainly improve geometric activity at high current densities by increasing accessible sites, improving mass transport, and promoting bubble detachment [103, 106, 133, 134, 135]. Accordingly, different strategies should adopt matched evaluation metrics: intrinsic effects need to be assessed in combination with electrochemical active surface area (ECSA) or site‐normalized activity; engineering effects should focus on high‐current polarization and mass transport; and stability should be judged by combining structural evolution and metal dissolution [136]. For Ni‐based alkaline systems, interference from trace Fe in KOH and from Ni foam substrates should also be ruled out [137]. From the perspective of intrinsic activity, Ce doping and regulation of CeO_2_ heterointerfaces are the most direct. After Ce enters the transition metal oxide lattice, it can alter the electronic states of neighboring metal centers through Ce‐O‐M bonding, 4f electron buffering, and local O_v_ [78, 119, 120, 138]; however, excessively high Ce content may cause precipitation of a separate CeO_2_ phase, so extended X‐ray absorption fine structure (EXAFS) or atomic‐resolution imaging is required to confirm actual substitution. Heterointerfaces can improve OER performance through interfacial charge transfer, OH^−^ enrichment, and active‐phase reconstruction [50, 85, 97, 127, 139, 140], but excess CeO_2_ may cover active sites and increase resistance, indicating that the effective interface proportion is more critical than simply increasing the CeO_2_ content [141, 142]. O_v_ engineering also has an optimal window: moderate O_v_ is beneficial for regulating Ce^3+^/Ce^4+^ and adsorption behavior, whereas excessive defects may weaken lattice stability, and some O_v_ may be refilled under strongly anodic potentials [112, 130, 131, 132]. The operating environment further determines strategy priority. In alkaline media, Ce doping, controlled O_v_, and heterointerfaces that promote reconstruction are more suitable for addressing intrinsic kinetic limitations; when entering high‐current‐density operation, hierarchical porous structures, self‐supported arrays, or in situ electrodeposition should be combined to improve mass transport, charge transport, and bubble release [97, 103, 106, 133, 134, 135]. In acidic OER, stability takes precedence over further increasing defects; therefore, CeO_2_ is more suitable as a stable support for Ir/Ru or as a solid‐solution component, where metal‐support interactions, Ir‐O‐Ce bonding, and electron buffering suppress overoxidation and dissolution [99, 100, 111, 121, 143, 144, 145]. For seawater OER, OER selectivity and resistance to Cl^−^ corrosion should be prioritized; CeO_2_ heterointerfaces and selective CeO_x_ overlayers are more targeted [86, 146], but an overlayer that is too thick will increase mass transport resistance. Therefore, there is no single optimal strategy for CeO_2_‐based OER catalysts that applies to all conditions. On this basis, we provide a critical comparison of the major design strategies in Table 1 and summarize their applicable conditions. A more rational design should be selected according to the actual rate‐limiting step: when intrinsic kinetics are insufficient, doping, controlled O_v_, and strongly coupled interfaces should be prioritized; for high‐current operation, porous structures and self‐supported electrodes should be emphasized; in acidic environments, stable supports or solid solutions should be prioritized; and in seawater environments, Cl^−^‐resistant interfaces and protective layers should be emphasized. At the same time, Ce content, interface proportion, and O_v_ concentration generally each have an optimal range, beyond which site dilution, increased resistance, or decreased structural stability may occur [130, 141, 142].

**TABLE 1:** Critical comparison and applicable conditions of major design strategies for CeO_2_‐based OER electrocatalysts.

Strategy (CeO_2_ configuration)	Primary lever	Primary performance bottleneck addressed	Most suitable conditions	Main limitations / required verification	Ref.
Ce‐doped host lattice (including atomically dispersed Ce)	Ce‐O‐M bonding, 4f‐electron buffering, local O_v_	Intrinsic activity / reconstruction regulation	Alkaline media; (oxy)hydroxide and spinel hosts	Limited Ce solubility; high loading may form a separate CeO_2_ phase. True substitution should be verified by EXAFS or atomic‐resolution imaging; Ce migration or dissolution during operation should also be examined.	[78, 119, 120]
CeO_2_ heterointerface (nanoparticles, islands, nanoheterojunctions)	Interfacial charge transfer, OH^−^ enrichment, reconstruction regulation	Intrinsic activity + interfacial stability	Alkaline media and seawater; S/P/Se‐based precatalysts	Performance depends on effective interfacial area; excessive CeO_2_ may block active sites and increase resistance. Morphology‐ and ECSA‐induced apparent gains should be excluded.	[50, 85, 97, 127, 139, 140]
CeO_2_ as a support (noble‐metal clusters, single atoms)	Metal‐support interaction, anchoring, electronic buffering	Long‐term stability/noble‐metal utilization	Acidic Ir/Ru (PEM) systems; single‐atom catalysts	Low CeO_2_ conductivity; Ce stability and metal anchoring should be verified at high anodic potentials in acid; carbon conductive additives may corrode under OER potentials.	[111, 130, 143, 145]
Ce‐containing solid solution / lattice strain	Ir‐O‐Ce bonding, electronic buffering, strain regulation	Acidic intrinsic activity + stability	Acidic OER with reduced noble‐metal loading	May be metastable and undergo phase separation during operation; single‐phase character and local coordination should be confirmed after operation or under operando conditions.	[99, 100, 121]
CeO_x_ protective / selectively permeable overlayer	Physical and chemical barrier, Cl^−^exclusion, local ion selectivity	Corrosion resistance / OER selectivity	Seawater and impurity‐containing water; protection of unstable active phases	An optimal coating thickness is required; overly thick or highly crystalline layers cause mass‐transfer losses and must balance Cl^−^ shielding with OH^−^ transport.	[86, 146]
O_v_ engineering (reduction, doping, etching)	O_v_/Ce^3+^ density, local electronic structure, adsorption regulation	Intrinsic activity	Multiple electrolytes; usually combined with other strategies	O_v_ may be refilled under anodic potentials and its effect is non‐monotonic. O 1s XPS alone is insufficient to prove O_v_; EPR, Raman, or XAS should be combined.	[112, 130, 131, 132]
Morphology / hierarchical porosity / self‐supported electrode	ECSA, mass transport, bubble release, binder‐free contact	High‐current‐density engineering performance	High‐current‐density and electrolyzer‐relevant operation	Activity advantages may diminish after ECSA normalization; contributions from Ni foam and Fe impurities in electrolyte should be excluded, and scalability should be assessed.	[103, 106, 133, 135]

## 3 Mechanistic Roles of CeO_2_ in OER

From the perspective of synergistic atomic and electronic structure modulation, the multifunctional catalytic characteristics of CeO_2_‐based electrocatalysts for the OER are fundamentally rooted in the dynamic evolution of Ce^3+^/Ce^4+^ redox coupled with O_v_, the reversible redox switching governed by the unique 4f electron configuration, and the consequent driving forces for interfacial reconstruction (Figure 7). At the atomic scale, abundant O_v_s within the CeO_2_ lattice not only serve as active sites that directly participate in the reaction pathway by finely tuning the distribution of adsorption free energies of oxygenated intermediates to lower the reaction barrier, but also induce an asymmetric redistribution of local charge density, thereby potentially triggering lattice oxygen oxidation mechanisms (LOM) that are kinetically more favorable. Concomitantly, the unique 4f electron configuration of cerium allows it to shuttle easily between Ce^3+^ and Ce^4+^, giving rise to what is known as a “d‐p‐f gradient orbital coupling” effect. Consequently, CeO_2_ operates as an electronic buffer: it continually adjusts the d‐band center position and the electron filling state of neighboring active metals. This behavior stabilizes centers in a high oxidation state and, at the same time, promotes rapid interfacial charge movement. Separately, O_v_ capture OH^−^ preferentially, which provides both thermodynamic and kinetic impetus for dynamic surface reconstruction. This impetus drives the targeted and accelerated transformation of hydroxide precursors into the catalytically superior oxyhydroxide phase. As the conversion proceeds, well‐defined heterointerfaces form between the two phases; these interfaces cooperatively refine the migration pathways and the adsorption/desorption characteristics of oxygen species. It should be noted that the above intrinsic properties provide a common mechanistic basis for different Ce configurations, but their dominant role still depends on the structural identity and spatial location of Ce species in the catalytic system [147]. When CeO_2_ acts as a cocatalyst, it mainly functions through charge redistribution at heterointerfaces, adsorption modulation, and promotion of the reconstruction of neighboring active phases, while CeO_2_ itself is not necessarily the main OER active center [93, 147]; when it acts as a support, in addition to interfacial regulation, it can also improve structural stability by anchoring and stabilizing active species and inhibiting overoxidation and dissolution [121]. In contrast, when Ce is incorporated into the host lattice in a doped form, its role arises more from charge rearrangement, lattice distortion, and defect modulation caused by local Ce‐O‐M coordination, thereby altering the local electronic structure of the host material [119]; whereas when CeO_2_ serves as the main component or continuous matrix, its Ce^3+^/Ce^4+^ redox and O_v_‐related defect chemistry participate more directly in the regulation of the entire catalytic system [121]. Therefore, although different configurations share a common intrinsic physicochemical origin of Ce, their dominant action pathways exhibit pronounced configuration dependence [93, 119, 121, 147]. In essence, the structural and chemical features of CeO_2_‐based systems are brought into alignment across atomic, electronic, and mesoscopic length scales, a multiscale coherence that serves as the theoretical basis for a concerted improvement of intrinsic activity and long‐term durability in the oxygen evolution reaction.

**FIGURE 7:**
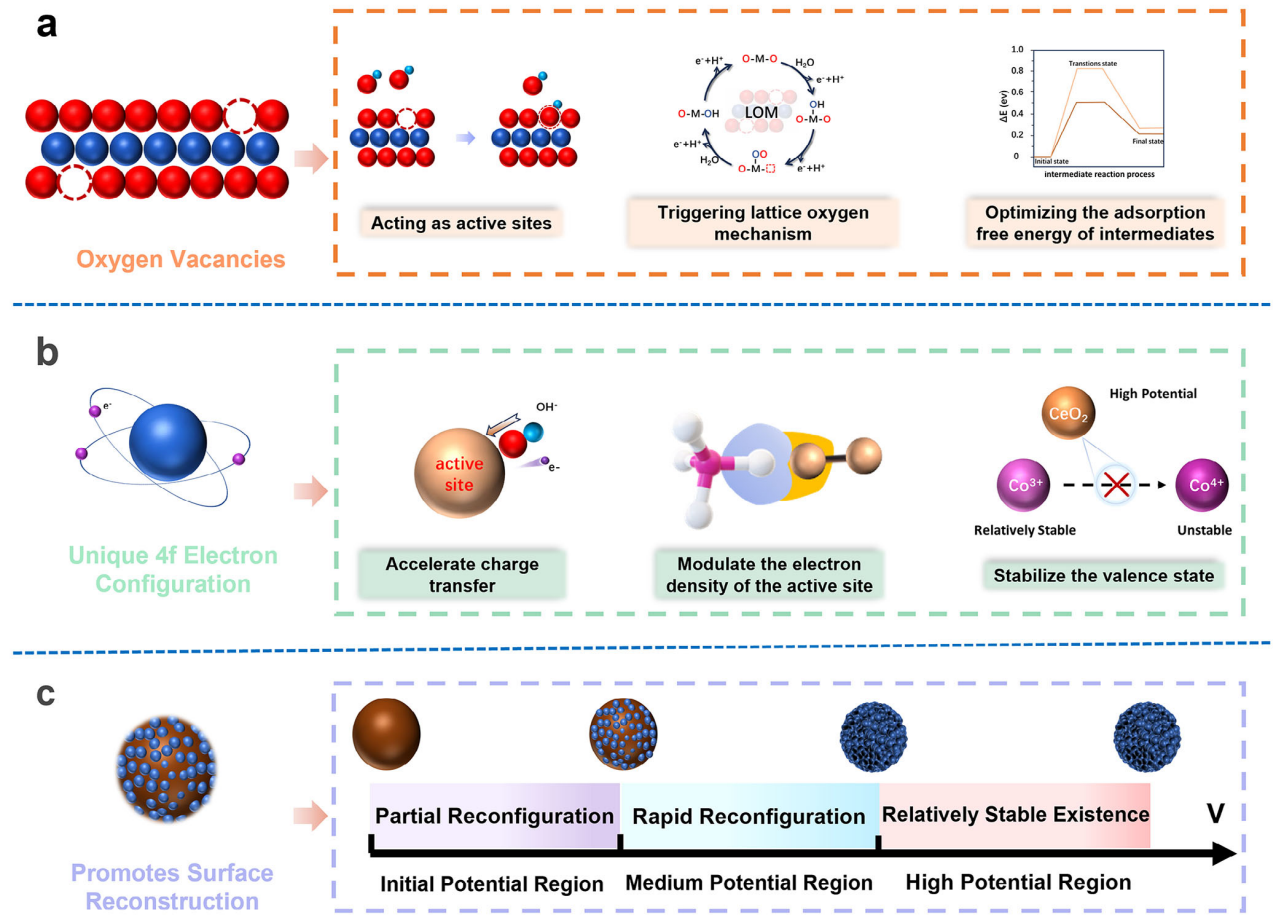
The multifunctional regulation mechanism of CeO_2_ in the OER catalyst system.

### 3.1 O_v_ Generation

O_v_s in CeO_2_‐based OER catalysts have long drawn attention as a central theme in electrocatalysis because their dynamic behavior is so tightly coupled to catalytic performance. As shown in Figure 8a–e, the fluorite structure of CeO_2_ confers a remarkable oxygen‐storage capacity, while the reversible Ce^3+^/Ce^4+^ redox couple facilitates the formation and stabilization of O_v_s through coupled charge compensation. In addition to the generation of O_v_ coupled with Ce^3+^/Ce^4+^ redox cycle, it has been observed that surface O_v_ can dynamically evolve through thermodynamically driven surface reconstruction. In a reducing atmosphere (e.g., CO), surface oxygen atoms desorb preferentially, leaving behind vacancies, while nearby Ce atoms migrate to relieve lattice strain. Under oxidizing conditions (e.g., CO_2_), the vacancies are sealed again by reverse oxygen refilling, a process intimately linked to the Ce^3+^→Ce^4+^ valence transition (Figure 8f) [148]. The spatial distribution of these vacancies, however, is far from uniform. Previous data show that the (111) facet is markedly more stable due to its higher oxygen coordination number, which translates into a higher vacancy formation energy, whereas the (100) and (110) planes tend to arrange vacancies into periodic superstructures (Figure 8g) [149]. Such crystal‐plane selectivity offers a rationale for vacancy engineering: by deliberately tuning the synthesis parameters, one can generate O_v_s‐rich surfaces that deliver substantially boosted catalytic activity. Within the OER, O_v_ sites can serve directly as active centers, adjusting the adsorption free energies of oxygenated intermediates and thereby lowering the reaction barriers [150, 151, 152]. A case in point is CeO_2_‐decorated Ni_3_S_2_, where O_v_‐induced reconstruction of a NiOOH interface, which markedly enhances OH^−^ adsorption and cuts the OER overpotential (Figure 8h–i) [50]. In contrast to its role as a cocatalyst, complementary DFT calculations reveal that the CeO_2_ surface preferentially forms O_v_, thereby inducing local charge redistribution and a mixed Ce^3+^/Ce^4+^ valence state. This electronic perturbation, working in concert with interfacial electron transfer across the heterojunction, fine‐tunes the binding of critical LOM intermediates and ultimately brings about a pronounced reduction in the O_2_ desorption energy barrier [59]. Notably, O_v_ concentration can also be experimentally adjusted; tuning it to an appropriate level further enhances OER activity (Figure 8j–l) [130]. The O_v_ in CeO_2_‐based OER catalysts serves both as providers of active sites and regulators of structural evolution. Precise modulation of O_v_ enables synergistic optimization of the catalyst's electronic structure and reaction kinetics, providing new insights for designing efficient and stable non‐noble metal OER catalysts.

**FIGURE 8:**
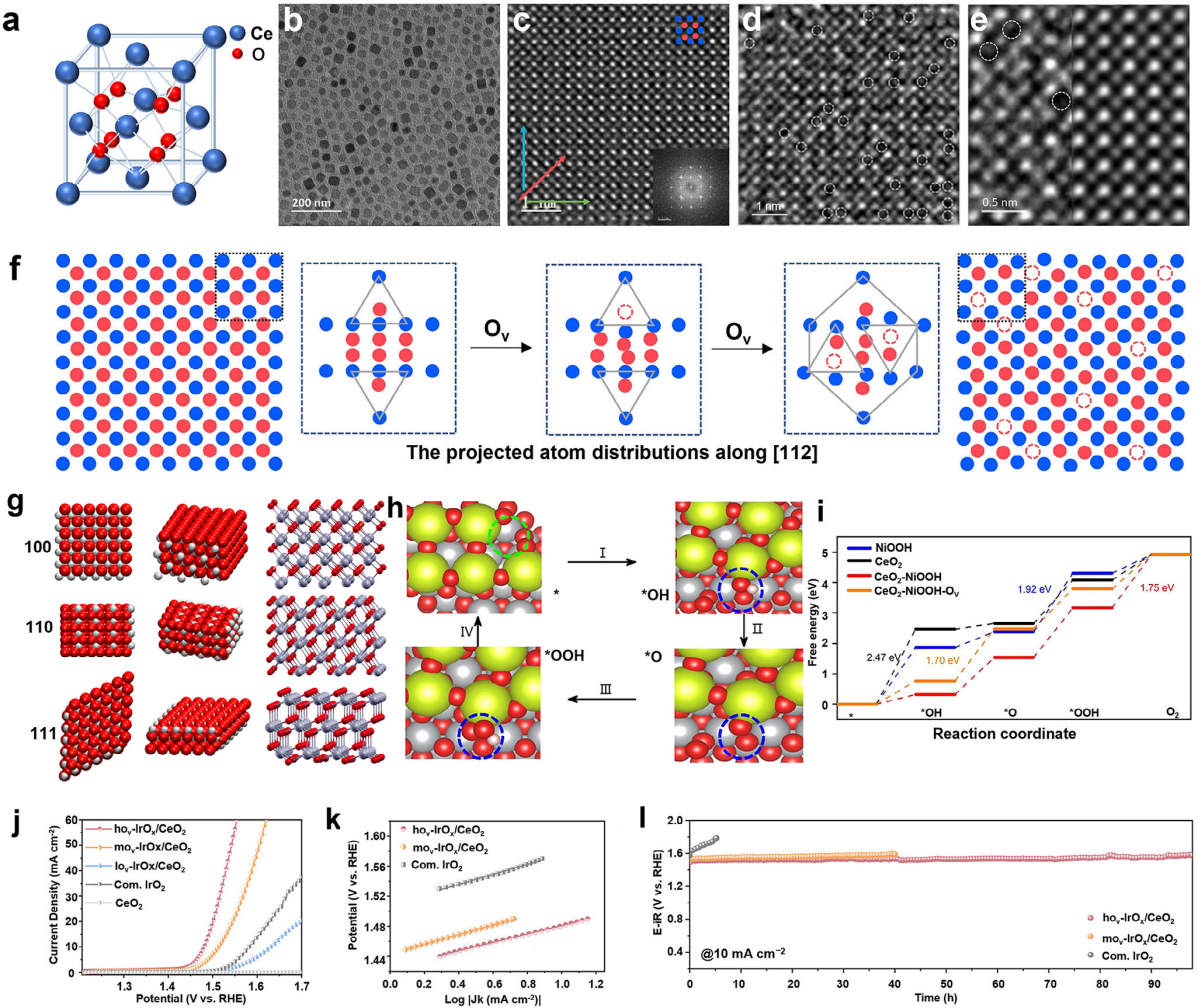
(a) Crystal structure of CeO_2_. (b) Low‐resolution TEM image of CeO_2_ nanocubes, (c) HRTEM image of CeO_2_ nanocubes, (d) HRTEM image of CeO_2_ nanocubes with O_v_s, (e) Locally magnified image derived from (d) and its corresponding inverse fast Fourier Transform (IFFT) pattern, (f) Schematic illustration of O_v_ formation in CeO_2_ nanocubes. Reproduced with permission [148]. Copyright 2023, Elsevier. (g) Top, side, and perspective views of CeO_2_ crystallographic facets: 100, 110, and 111, cerium and oxygen ions are represented by grey and red spheres, respectively. Reproduced with permission [149]. Copyright 2025, American Chemical Society. (h) Schematic of the OER reaction pathway, showing adsorbed intermediates on the CeO_2_‐NiOOH‐O_v_ surface. Grey, yellow, red, and white spheres denote Ni, Ce, O, and H atoms; green circles indicate O_v_s, (i) Free energy diagrams for OER pathways on CeO_2_, NiOOH, CeO_2_‐NiOOH, and CeO_2_‐NiOOH‐O_v_ surfaces. Reproduced with permission [50]. Copyright 2022, American Chemical Society.(j–l) Oxygen evolution performance of IrO_x_/CeO_2_ with varied O_v_ concentrations: High O_v_ concentration (hov‐IrO_x_/CeO_2_), Medium O_v_ concentration (mov‐IrO_x_/CeO_2_), Low O_v_ concentration (lov‐IrO_x_/CeO_2_) (j) Linear sweep voltammetry (LSV) curves, (k) Tafel plots, (l) Chronopotentiometric curves at 10 mA cm^−2^. Reproduced with permission [130]. Copyright 2023, American Chemical Society.

### 3.2 Electronic Structure Modulation

CeO_2_, owing to its unique 4f electronic configuration and tunable redox properties, can be modulated through strategies such as doping, heterostructure construction, and strain engineering to tailor its electronic structure, thereby significantly optimizing OER activity and stability [147, 153]. This section systematically summarizes the key mechanisms and recent advances in the modulation of the electronic structure of CeO_2_. Unlike Section 2.4, which focuses on interfacial configurations and structural roles, this section emphasizes the electronic effects that emerge after these structures form, including charge transfer, orbital coupling, and changes in adsorption energy [114, 129]. In terms of doping engineering, CeO_2_ possesses a fluorite‐type cubic crystal structure, and the ionic radii of Ce^3+^ and Ce^4+^ are 1.14 and 0.97 Å, respectively. If CeO_2_ is introduced as a dopant into other materials, the larger ionic radius induces lattice distortion, thereby reducing the structural stability. Moreover, the O_v_ occupied core functionality of CeO_2_ may be isolated. Therefore, more homogeneous doping is achieved by incorporating Ce in its ionic form, where the electronegativity difference between Ce and the host catalyst metal and the unique 4f orbital characteristics enable precise electronic regulation: because Ce possesses significantly lower electronegativity than transition metals, it tends to donate electrons to adjacent metal sites and coordinated oxygen, thereby avoiding excessive oxidation of the active metal into easily dissolved high‐valence species (Figure 9a–f) [119, 138]. Simultaneously, through the construction of Ce(4f)‐O(2p)‐Co(3d) gradient orbital coupling units by introducing single‐atom Ce, the unoccupied Ce 4f orbitals serve as an electronic buffer that provides electronic compensation to the active center during OER. This mechanism suppresses the over‐oxidation of Co‐O bonds, preserves Co‐O covalency, and optimizes the occupancy of Co‐3d‐e_g_ orbitals, thereby balancing the adsorption strengths of oxygen intermediates and significantly enhancing catalytic activity [78]. The optimization of the electronic structure concurrently modulates the d‐band center of the host catalyst, further reducing the Gibbs free energy of the OER rate‐determining step. For instance, in CeO_2_‐CoOOH, the free energy of the rate‐determining step in the AEM pathway decreases substantially [154]. It has also been demonstrated that employing CeO_2_ as a support retains its unique O_v_ and Ce^3+^/Ce^4+^ redox couple while allowing the introduction of other dopants for synergistic catalysis, further optimizing OER performance [155]. For example, doping Ni into CeO_2_ ultrathin nanosheet assemblies (NSAs) significantly increases the Ce^3+^ fraction; the enrichment of Ce^3+^ enhances the adsorption of oxygenated intermediates such as OH^−^. Concurrently, heteroatom doping induces more O_v_s, which lower the deprotonation energy barrier, improve electrical conductivity, and increase the number of active sites, thereby synergistically optimizing the oxygen evolution performance [65]. As a cocatalyst, the most extensively studied strategy for CeO_2_ is the construction of heterojunctions. CeO_2_‐based heterojunctions can encompass virtually all electronic modulation mechanisms. Because differences in work function and Fermi level commonly exist between distinct components, formation of a heterointerface can induce directional charge transfer and establish a built‐in electric field, thereby altering the local electronic states of adjacent active sites [114, 129]. For example, Kelvin probe force microscopy (KPFM) analysis of a NiTe/CeO_2_ heterostructure shows that the work functions of CeO_2_ and NiTe are 4.26 and 4.49 eV, respectively, yielding a work‐function difference of 0.23 eV that drives electron transfer from CeO_2_ to NiTe until Fermi‐level alignment and forms a built‐in electric field at the interface. Consequently, the electron density increases on the NiTe side and decreases on the CeO_2_ side, effectively modulating the interfacial electronic structure; the resulting positive charge accumulation on the CeO_2_ side enhances OH^−^ adsorption [114]. As shown in Figure 9g–i, similar to the single‐atom Ce doping mechanism, CeO_2_ can modulate the electronic structure of the host catalyst through the heterointerface, altering the d‐band center and charge distribution of active sites and accelerating charge transfer [52, 85, 156, 157, 158]. At the interfacial region where CoO and CeO_2_ are in close contact, strong electronic coupling reconstructs the electronic structure and generates highly active sites [127]; CeO_2_ acting as a surface modifier can also facilitate interfacial electron transfer [129]. In addition, in the CeO_2_/Ce‐Co_3_O_4_ system, the interfacial built‐in electric field and interatomic charge transfer through the Co‐O‐Ce configuration synergistically regulate electron localization and the d‐band center at Co sites: the Bader charge further decreases from −1.29 eV for Co_3_O_4_ and −1.27 eV for Ce‐Co_3_O_4_ to −0.85 eV, the d‐band center upshifts from −1.993 to −1.645 eV, and the RDS energy barrier decreases from 1.55 eV to 1.44 eV, thereby enhancing OER activity [129]. Meanwhile, it effectively tunes the electronic structure of the active sites, optimizing the adsorption and desorption energies of oxygenated intermediates (^*^OH, ^*^O, ^*^OOH), thus accelerating the O‐O bond formation step (^*^O → ^*^OOH) and enhancing the overall OER electrocatalytic efficiency (Figure 9j) [106, 159, 160]. During the OER process, the unique Ce^3+^/Ce^4+^ reversible redox couple in CeO_2_ acts as an efficient electronic mediator, lowering the energy barrier for valence‐state transitions of metal ions in the host catalyst and triggering the conversion of Co or Ni species into highly active valence states at lower anodic potentials [52, 85, 157]. Beyond these widely investigated regulation strategies, there are also fewer common approaches. For instance, in situ grown CeO_2_ nanoparticles induce interplanar spacing compression while modulating the electronic structure through interfacial charge transfer, thereby altering the oxygen evolution reaction pathway [100]. When designing CeO_2_ to enhance OER activity through electronic structure modulation, it is highly desirable to comprehensively exploit the above‐mentioned characteristics to simultaneously improve both activity and stability.

**FIGURE 9:**
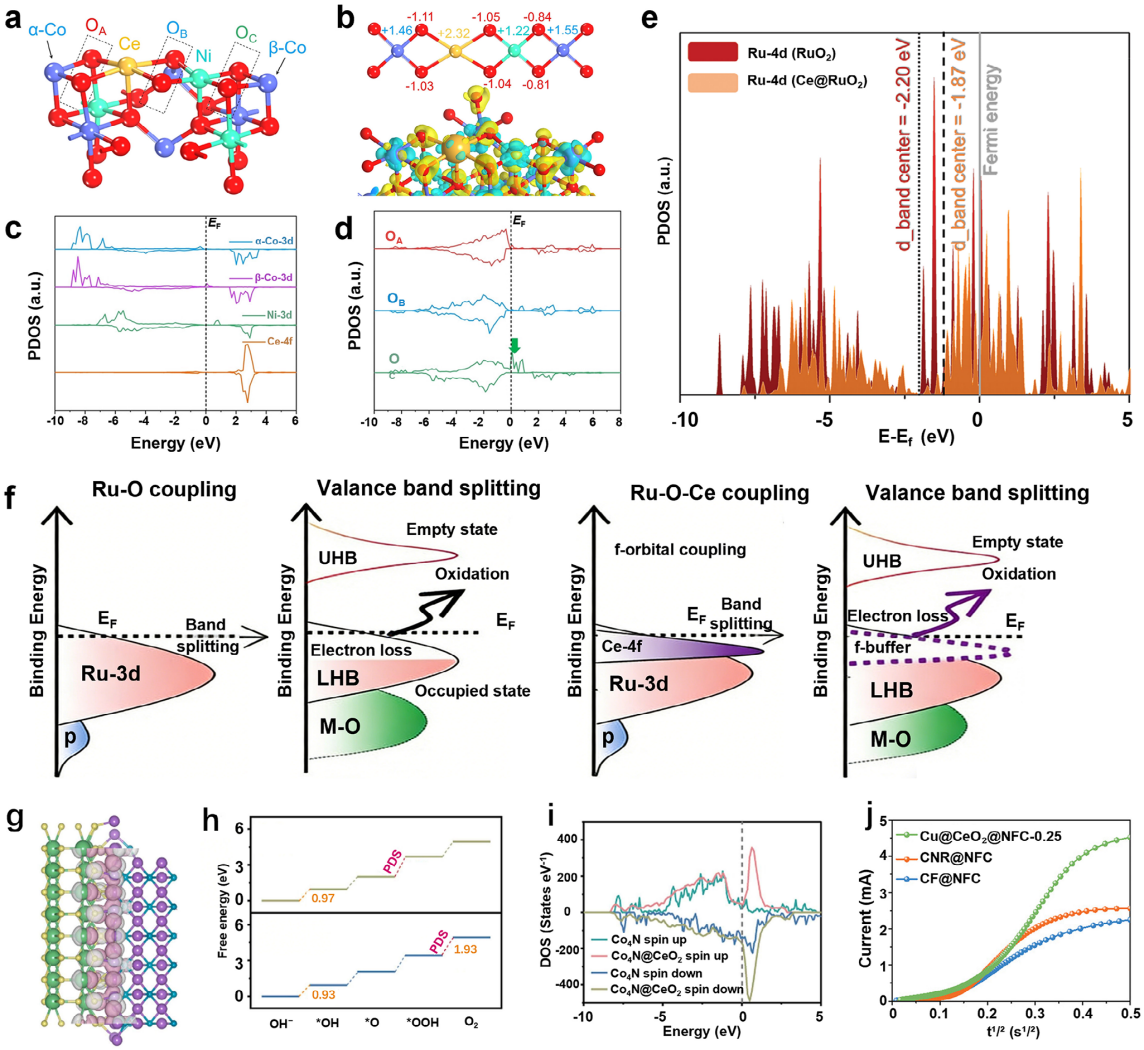
(a–f) Electronic modulation of OER catalysts by Ce doping. (a) Schematic model of Ce‐doped NiCO_2_O_4_ nanosheets. (b) Charge density difference plot based on Bader charge population, where yellow regions indicate charge accumulation and cyan regions indicate charge depletion. (c,d) Projected density of states (PDOS) of surface atoms from (a). Reproduced with permission [119]. Copyright 2024 John Wiley and Sons. (e) Construction of d‐p‐f gradient orbitals coupled with f‐band electron buffers in Mott‐Hubbard splitting of Ce@RuO_2_/CoNC. (f) Ru 4d partial density of states in RuO_2_ and Ce@RuO_2_ catalysts (dark red corresponds to RuO_2_ PDOS, orange corresponds to Ce@RuO_2_ PDOS). Reproduced with permission [138]. Copyright 2024 John Wiley and Sons. (g–i) Electronic modulation of OER catalysts by constructing CeO_2_ heterojunctions. (g) Charge density difference distribution at the Co_4_N@CeO_2_ interface (light gray: electron accumulation; light pink: electron depletion). (h) Free energy changes during H_2_O dissociation. Surface Co sites in Co_4_N and interfacial Ce sites in Co_4_N@CeO_2_ were selected as adsorption sites for H_2_O. (i) Total DOS of Co_4_N and Co_4_N@CeO_2_. The Fermi level is shifted to zero. Reproduced with permission [156]. Copyright 2023 Elsevier. (j) Enhanced mass transfer at the ceria heterointerface, chronoamperometric measurement of oxygen diffusion rate. Reproduced with permission [160]. Copyright 2020 John Wiley and Sons.

### 3.3 Surface Reconstruction

For OER electrocatalysts, elucidating the origin of their high activity and investigating surface reconstruction and dynamic evolution processes are crucial. As mentioned in the previous subsection, CeO_2_‐based heterointerfaces can drive the in situ reconstruction of primary catalysts [161, 162]. The dynamic surface reconstruction process exhibits multi‐level regularity across temporal and spatial scales. This section systematically elucidates the role of CeO_2_ within composite structures and interfaces in regulating OER kinetics from a spatiotemporal perspective, governing dynamic evolution with emphasis on interfacial coupling and surface reconstruction (Figure 10a–d). It should be noted that CeO_2_‐induced reconstruction is still driven by the applied potential; compared with unmodified catalysts, interfacial coupling modifies the pathways of metal oxidation, deprotonation, and local coordination evolution, rather than constituting a mechanism independent of the applied potential [93, 157]. This interfacial regulation is illustrated by representative Ni‐based systems, in which interfacial charge redistribution modifies the initial Ni oxidation response at lower polarization potentials [163]. For example, in CeO_2_/NiO, electron transfer from NiO to CeO_2_ increases the Ni^3+^ content; the study links this higher Ni oxidation state to enhanced Ni‐O covalency [150]. As the potential rises and polarization proceeds, this modified electronic environment facilitates Ni oxidation; together with OH^−^ adsorption and surface deprotonation, it favors conversion of the hydroxylated surface into NiOOH, helping explain its appearance at lower potentials [50, 163]. Once the active surface develops, interfacial coupling can continue to influence Ni‐O bonding and stabilize the Ni oxidation state during OER [150]. CeO_2_ therefore affects both active‐phase formation and the electronic environment of the reconstructed interface, without necessarily producing a different phase: Ce‐containing Ni‐Fe‐Co oxides still form metal oxyhydroxides under operating conditions [163], while post‐OER signals in CeO_2_‐modified Ni_3_N have been assigned to β‐NiOOH [164]. A lower formation potential alone does not establish a faster reconstruction rate. The pathway also remains system‐dependent: in acidic Co_3_O_4_/CeO_2_, CeO_2_‐induced shortening of Co‐O bonds modifies the Co‐O bonding environment, which facilitates the formation of OER‐active Co^4+^ species and thereby bypasses the pronounced reconstruction process associated with charge accumulation [157]. The O_v_ in CeO_2_ acts as a catalytically active defect site. The O_v_ forms upon the generation of interface and the exposure of surrounding Ce^3+^ ions (originally Ce^4+^) [97, 103]. The low‐coordination state of Ce^3+^ enhances its Lewis acidity, enabling preferential adsorption and enrichment of OH^−^ ions in the solution through electrostatic interactions, thereby providing a driving force for the surface reconstruction of active surfaces, such as transition metal oxides [50]. The strong d‐p‐f gradient orbital coupling driven by interfacial interactions between CeO_2_ and transition metals is another critical factor that triggers surface reconstruction. The role of CeO_2_ varies across different systems and environments. When CeO_2_ is in an oxidized state (Ce^4+^‐dominant), it tends to accept electrons from low‐oxidation‐state metals (e.g., Co^2+^). In O_v_‐rich CeO_2‐x_, where the Ce^3+^ ratio increases, CeO_2_ tends to donate electrons to high‐oxidation‐state metals, thereby accelerating the reconstruction process [97, 127]. As shown in Figure 10e–i, during the initial OER stage, the O_v_ and electron transfer effects of CeO_2_ initiate localized reconstruction. CeO_2_‐modified transition metal compounds exhibit peaks of active phases at lower potentials [85]. As the potential increases to the medium‐potential region, rapid reconstruction occurs, forming highly active structures at the interface between transition metals and CeO_2_. The reconstructed active interface stabilizes over time, with denser interfacial structures [97]. The CeO_2_ modification layer preferentially induces oxidation of transition metals on the primary catalyst surface through interfacial interactions. The internal structure gradually transforms into amorphous CoOOH after OER, while the bulk retains part of its initial structure (Figure 10j–l) [85]. After reconstruction, CeO_2_ does not merely play an initiation role: in situ X‐ray absorption spectroscopy (XAS) of CoO_x_/CeO_2_ shows that the Co valence changes with potential while the Ce L3‐edge remains essentially unchanged; combined with changes in surface Co/O structure, this supports that CeO_2_ continuously regulates the electronic structure of the active interface rather than proving that Ce directly participates in the oxygen evolution cycle [93]. In addition, a selectively permeable CeO_x_ layer on NiFeO_x_ can suppress Fe leaching while allowing OH^−^ and O_2_ to pass through without reducing its OER activity, providing another mechanism for continuously stabilizing the active components; however, this protective‐layer effect cannot be directly equated with the role of all CeO_2_ heterointerfaces [165]. Therefore, participation in interfacial regulation after reconstruction should be clearly distinguished from acting as a direct reaction center, and the reversible valence change of Ce and the defect cycle still require in situ evidence from the corresponding systems. CeO_2_ not only accelerates reconstruction but also maintains interfacial stability through dynamic equilibrium. During OER, filling of O_v_ by OH^−^ or O species oxidizes Ce^3+^ to Ce^4+^, whereas regeneration of O_v_ reduces Ce^4+^ back to Ce^3+^ [112]. This cyclic process stabilizes O_v_ concentration during prolonged testing, ensuring sustained reconstruction [97]. Differences exist in the reconstruction pathways of transition metals across systems. For example, Ni phosphides and sulfides modified with CeO_2_ are more prone to fracture, releasing S/P anions to form O_v_‐rich NiOOH interfaces. This indicates more thorough reconstruction in sulfide/phosphide systems [46]. Nevertheless, CeO_2_‐mediated promotion of transition metal OER catalyst reconstruction follows universal mechanisms. As a co‐catalyst, CeO_2_ significantly accelerates surface reconstruction of transition metal catalysts through O_v_‐induced OH^−^ adsorption, interfacial electron transfer, and dynamic O_v_ replenishment, while optimizing the electronic structure and reaction pathways of the reconstructed interfaces.

**FIGURE 10:**
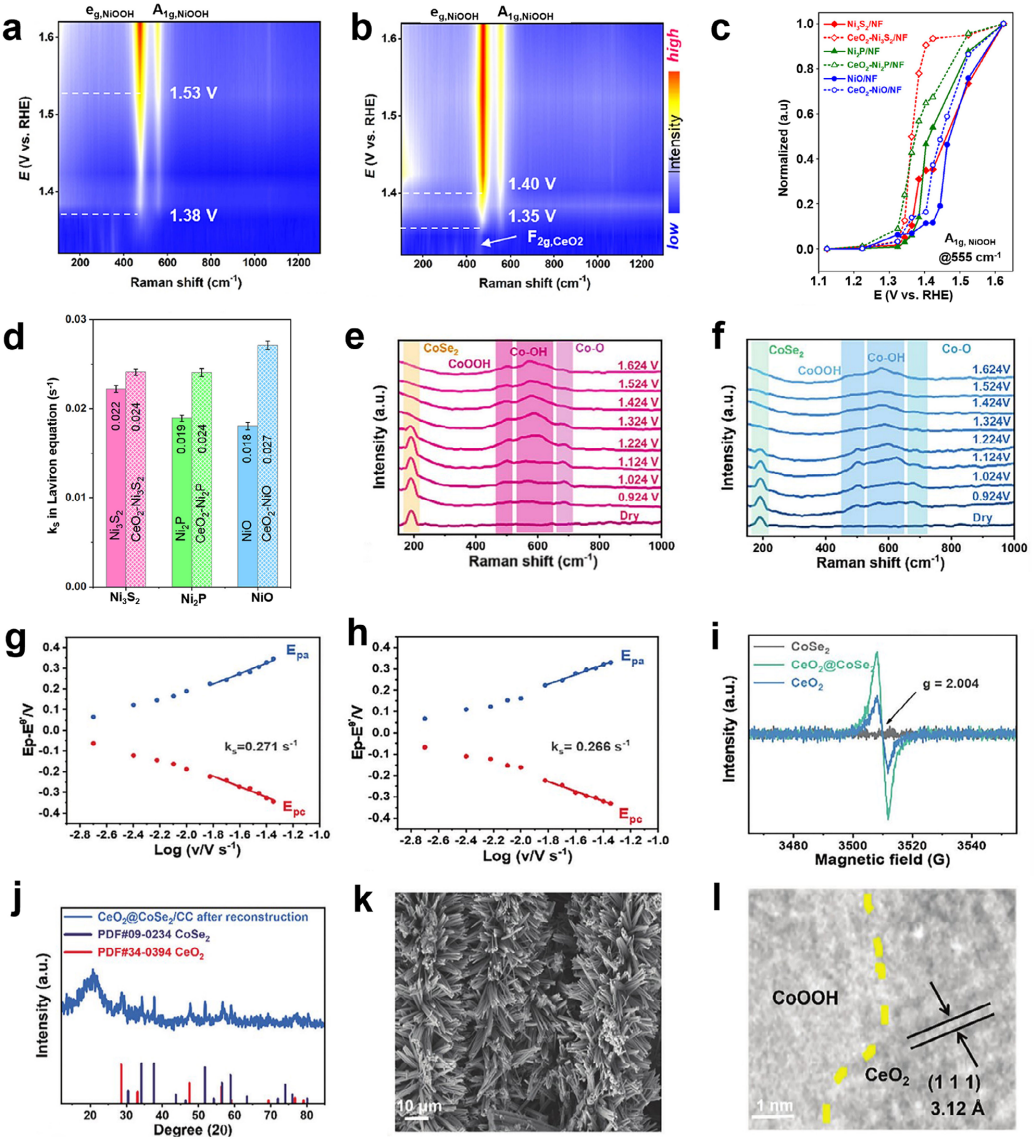
CeO_2_ shifts the surface reconstruction of Ni‐ and Co‐based precatalysts to lower potentials and modifies the structure of the reconstructed interface. (a,b) In situ Raman contour plots of (a) Ni_3_S_2_/NF and (b) CeO_2_‐Ni_3_S_2_/NF at different potentials (vs. RHE) in 1.0 M KOH; the E_g_ and A_1g_ bands of NiOOH (about 475 and 555 cm^−1^) appear at about 1.35 V with CeO_2_ compared with about 1.38 V without it, and the conversion is complete at about 1.40 V compared with about 1.53 V, while the F_2g_ band of CeO_2_ persists, showing that CeO_2_ is retained as a distinct phase. (c) Normalized Raman intensity at 555 cm^−1^ versus potential for sulfide, phosphide and oxide precatalysts with and without CeO_2_, showing that the active‐phase signal appears at lower potentials in all three systems. (d) Apparent electron‐transfer rate constants of Ni_3_S_2_/NF, Ni_2_P/NF, NiO/NF and their CeO_2_‐modified counterparts obtained by Laviron analysis at different scan rates. Reproduced with permission [50]. Copyright 2022 American Chemical Society. (e,f) In situ Raman spectra of (e) CoSe_2_/CC and (f) CeO_2_@CoSe_2_/CC, showing the loss of the CoSe_2_ band (about 190 cm^−1^) and the emergence of Co‐OH and Co‐O bands with increasing potential. (g,h) Laviron analysis of the Co redox couple of (g) CeO_2_@CoSe_2_/CC and (h) CoSe_2_/CC, from which the apparent electron‐transfer rate constants ks are obtained. (i) EPR spectra of CoSe_2_, CeO_2_@CoSe_2_ and CeO_2_; the O_v_‐related signal at g = 2.004 is strongest for CeO_2_@CoSe_2_, indicating enrichment of O_v_ at the interface. (j–l) Characterization of CeO_2_@CoSe_2_/CC after reconstruction: (j) XRD pattern, (k) SEM image showing that the nanoneedle array is retained, and (l) HRTEM image showing amorphous CoOOH adjacent to crystalline CeO_2_ (111) with a lattice spacing of 3.12 A. Reproduced with permission [85]. Copyright 2024 John Wiley and Sons.

### 3.4 Operando Evidence and the Controversy Over Lattice‐Oxygen Participation in CeO_2_

The preceding discussion demonstrates that CeO_2_ can continuously alter the local structure and reaction pathways of OER‐active phases through oxygen‐vacancy evolution, interfacial electronic regulation, and surface reconstruction. However, a central question remains unresolved: whether the lattice oxygen of CeO_2_ itself directly enters the OER catalytic cycle, or whether CeO_2_ mainly regulates the lattice‐oxygen activity of neighboring transition‐metal active phases through interfacial interactions. Because increased O_v_ concentration, changes in the Ce^3+^/Ce^4+^ ratio, pH dependence, and DFT predictions are largely indirect evidence, a rigorous distinction between these possibilities still requires isotope tracing combined with operando structural characterization. At present, ^18^O isotope labeling coupled with differential electrochemical mass spectrometry and online electrochemical mass spectrometry (DEMS/OLEMS) remains one of the most direct approaches for determining whether lattice oxygen enters evolved O_2_. Huang et al. detected ^16^O^18^O and ^18^O_2_ in an O_v_‐rich Fe_2_O_3_@CeO_2_ heterostructure, demonstrating lattice‐oxygen participation and showing that O_v_s preferentially formed on the CeO_2_ side [59]. In Ce‐doped CoTeO_3_, ^18^O‐DEMS together with in situ electron paramagnetic resonance (EPR) and Raman also supported defect evolution, surface reconstruction, and LOM characteristics [166]. Nevertheless, these results cannot be generalized as direct proof that fluorite CeO_2_ lattice oxygen universally enters O_2_: the former cannot spatially distinguish oxygen from the CeO_2_ and Fe_2_O_3_ sublattices, whereas the latter contains Ce as a dopant rather than an independent CeO_2_ phase. Moreover, residual H_2_^18^O, structural water, isotope exchange, and detection sensitivity can complicate DEMS‐based LOM assignment [167]. Existing operando spectroscopy further reveals how CeO_2_ regulates adjacent active phases. Operando HXAS of Fe‐CoO/CeO_2_ showed that CeO_2_ modifies the potential‐dependent oxidation behavior of Co and Fe [168], while in situ Raman of CeO_2_@NiS_x_/C_3_N_4_ revealed Ce‐promoted NiOOH formation and modulation of the interfacial water network [169]. In addition, operando X‐ray diffraction (XRD) of NiCeO_x_H_y_ showed that Ce incorporation facilitates the transformation toward γ‐NiOOH with exposed (003) facets, directly linking Ce‐induced structural evolution to the formation of the OER‐active phase [170]. Thus, XAS, Raman, and XRD provide complementary information on oxidation states, local coordination, and phase reconstruction, but none alone can determine the oxygen source in evolved O_2_. The information provided by representative operando/in situ techniques and their main limitations are summarized in Table 2. Overall, lattice‐oxygen participation in CeO_2_‐based OER systems is strongly configuration‐dependent. Current evidence confirms that CeO_2_ or Ce species can regulate defects, electronic structure, and reconstruction and can promote LOM in selected systems, but it remains insufficient to demonstrate universal, direct participation of CeO_2_ lattice oxygen. Future studies should combine selective ^18^O labeling and DEMS with operando X‐ray absorption fine structure spectroscopy (XAFS), Raman, and XRD, while EPR and low‐dose liquid‐phase transmission electron microscopy (TEM) provide complementary information on defect cycling and interfacial evolution. Only when oxygen origin, local structure, and electronic state are consistently correlated within the same catalyst can direct CeO_2_ lattice‐oxygen participation be distinguished from predominant regulation of neighboring active phases.

**TABLE 2:** Representative operando/in situ characterization methods for CeO_2_‐based OER systems, the information obtained, and their major limitations.

Characterization technique	Main information obtained	Representative evidence in Ce/CeO_2_ systems	Major limitations	Ref.
^18^O labeling + DEMS/OLEMS	Distinguishes ^16^O_2_, ^16^O^18^O, and ^18^O_2_ and traces the origin of oxygen in evolved O_2_	Lattice‐oxygen participation in Fe_2_O_3_@CeO_2_‐O_v_; ^18^O‐DEMS‐supported LOM in CeCoTeO_3_	Sublattice assignment is difficult in heterostructures; residual isotope‐enriched water, structural water, oxygen exchange, and detection sensitivity may cause misinterpretation	[59, 166, 167]
Operando XAFS/XAS	Tracks element‐specific oxidation states, coordination numbers, bond lengths, and local structure versus potential	Operando hXAS of Fe‐CoO/CeO_2_ showed that CeO_2_ alters Co/Fe oxidation processes	Element‐selective but spatially averaged; minority interfacial changes may be obscured and the oxygen source in O_2_ cannot be directly identified	[168]
In situ/operando Raman	Probes M‐O vibrations, active‐phase formation, surface reconstruction, and interfacial water structure	NiOOH formation/interfacial‐water modulation in CeO_2_@NiS_x_/C_3_N_4_; dynamic reconstruction in CeCoTeO_3_	Raman alone cannot prove LOM; peak overlap, fluorescence, laser heating, and O_2_ bubbles can interfere	[166, 169]
In situ/operando XRD	Tracks crystalline phase transitions, lattice parameters, strain, crystallinity, and average structural evolution under applied potential	Operando XRD of NiCeO_x_H_y_ revealed Ce‐facilitated formation of γ‐NiOOH with exposed (003) facets	Weakly crystalline/amorphous surface species may be difficult to resolve; bulk crystalline signals can mask thin reconstructed surface layers and time resolution is limited.	[170]
Electrochemical liquid‐phase TEM	Real‐time observation of particles, phase boundaries, dissolution/deposition, and nanoscale reconstruction	Widely used to monitor OER structural evolution; direct applications to CeO_2_‐based OER remain limited	Electron‐beam‐induced water radiolysis and the transport/local environment in miniature liquid cells may deviate from conventional electrolysis.	[171, 172]
In situ/operando EPR	Detects paramagnetic defects, trapped electrons, O_v_s, and radical intermediates	EPR confirmed O_v_s in Fe_2_O_3_@CeO_2_‐O_v_; in situ EPR tracked defect evolution in CeCoTeO_3_	Truly potential‐controlled CeO_2_‐OER studies remain scarce; aqueous microwave losses, conductive electrodes, weak signals, and complex spectral assignment limit quantification.	[59, 166, 173]

## 4 Performance of CeO_2_‐Based OER Catalysts in Different Electrolytes

The catalytic behavior of CeO_2_‐based electrocatalysts for the oxygen evolution reaction is likewise profoundly influenced by the electrolytic environment, with their structural adaptability and interfacial response mechanisms manifesting distinct functional pathways under varying pH and ionic strength conditions (Figure 11). Under alkaline conditions, what primarily unlocks the catalytic performance of CeO_2_ is atomic‐scale electronic‐state engineering combined with defect‑chemical design. Doping with heteroatoms, such as sulfur, nitrogen, or phosphorus, or constructing composite architectures with Co‐ or Ni‐based transition metals raises the O_v_ population and redistributes the Ce^3+^/Ce^4+^ ratio. This, in turn, tunes the adsorption energetics and desorption barriers of oxygenated intermediates at surface active sites, speeding up OER kinetics. Central to these strategies is a synergistic promotion of active‐facet exposure and charge‐transport properties, achieved through structural tuning that ranges from the mesoscopic down to the atomic level. In strongly acidic electrolytes, CeO_2_ rarely serves as the primary active phase. Rather, it exploits its electron‐buffering capacity and the reversible Ce^3+^/Ce^4+^ redox couple to dynamically modulate the valence distribution of active centers and to suppress the dissolution and leaching of metallic constituents at elevated potentials. This is typically accomplished by forming heterointerfaces or solid‐solution‐type coupled structures with noble‐metal components (e.g., Ir, Ru). Such stabilization markedly extends the catalyst's operational lifetime while preserving high intrinsic activity. The underlying stabilization mechanism is, in essence, a synergistic consequence of interfacial electronic reconfiguration and chemical‑environmental buffering. When the electrolyte environment shifts to high‐salinity, highly corrosive seawater systems, CeO_2_‐based electrocatalysts exhibit a distinctive adaptive interfacial micro‐environmental response. Consistent with hard‐soft acid‐base theory, OH^−^ species preferentially accumulate on the CeO_2_ surface, spontaneously creating a local alkaline reaction micro‐zone. At the same time, arising from the surface‑charge characteristics of CeO_2_, electrostatic repulsion effectively shields against nucleophilic attack by chloride ions. This repulsion suppresses the competing chlorine‑evolution side reaction while ensuring high Faradaic efficiency and long‐term operational stability of the OER process. Fundamentally, this effect represents a cross‑scale protective strategy governed by interfacial charge distribution and ion‑selective transport. To summarize, the functional behavior of CeO_2_ across diverse electrolytic scenarios stems from its structural plasticity and the tunability of its interfacial chemistry: it stabilizes active components through heterostructures, reshapes electronic states and reaction pathways via doping and defect engineering, and excludes corrosive species through surface‑chemical micro‐environmental exclusion. These cross‐scale mechanistic features collectively constitute the design foundation for CeO_2_‐based oxygen evolution electrocatalysts to deliver efficient, highly stable operation in multi‑scenario water electrolysis systems.

**FIGURE 11:**
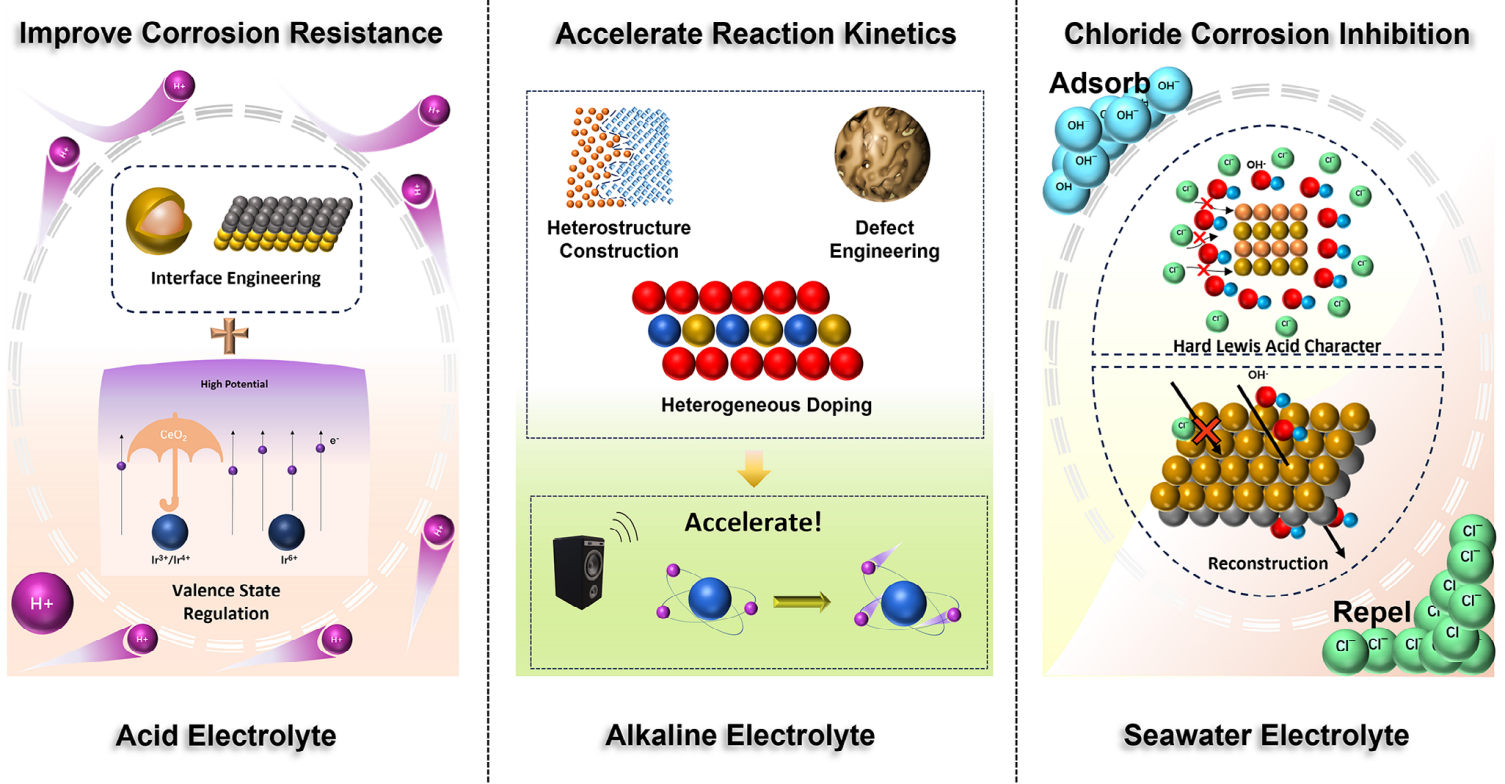
The role of CeO_2_ in the OER under different environments.

### 4.1 Alkaline Electrolytes

During water electrolysis, alkaline media pose a markedly lower corrosion risk to electrode and electrolyzer materials than acidic environments do. Running the process under acidic conditions, by contrast, forces the use of noble‐metal catalysts, such as platinum, iridium, and ruthenium, simply to withstand the aggressive corrosion, which drives up costs considerably. For this reason, alkaline water electrolysis remains the prevailing industrial route today. Against this backdrop, CeO_2_‐based electrocatalysts have drawn substantial research interest as highly active electrocatalysts for OER in alkaline electrolytes. Their O_v_ defects, reversible Ce^3+^/Ce^4+^ redox couples, and excellent oxygen storage/release capacity make them promising low‐cost components of composite OER catalysts that can reduce the reliance on noble‐metal catalysts (e.g., IrO_2_, RuO_2_) [174, 175, 176]. However, pure‐phase CeO_2_ often exhibits unsatisfactory OER performance, such as high overpotential and slow reaction kinetics, due to its intrinsic poor conductivity and limited exposed active sites [131]. To significantly enhance its catalytic performance, researchers have developed various effective modification strategies, mainly including heteroatom doping, constructing heterostructures with other functional materials, morphology and defect engineering, and designing self‐supporting electrodes. As shown in Figure 12a–c, in terms of heteroatom doping, the introduction of non‐metallic elements (e.g., S, N, P, B) and transition metal elements (e.g., Fe, Ni) is a key means to modulate the electronic structure of CeO_2_ and increase O_v_ concentration, thereby enhancing activity [94, 131, 132, 177]. Representative non‐metal doping is S‐doped CeO_2_ (S‐CeO_2_), which requires an overpotential of only 190 mV to reach 10 mA cm^−2^ in 1 M KOH, with a Tafel slope of 83 mV dec^−1^, and demonstrates excellent stability exceeding 20 h; theoretical calculations indicate that S doping introduces impurity energy levels near the O_2_/H_2_O energy levels, promoting electron transfer and optimizing the adsorption energy of oxygen‐containing intermediates [131]. Transition metal doping, particularly Fe doping and Ni/Fe co‐doping, can induce highly disordered structures and numerous O_v_s in CeO_2_ [94, 132]. In transition metal‐doped and co‐doped CeO_2_ nanostructures, the O_v_ concentration increases markedly; concurrently, modulation of the electronic orbital structure facilitates the LOM pathway (Figure 12d–f) [94, 139]. In constructing heterostructures, compounding CeO_2_ with other metal oxides (e.g., CoO, NiO), phosphides (e.g., CoP), noble metal oxides (e.g., RuO_2_), or carbon materials (e.g., graphene, reduced graphene oxide rGO) can synergistically enhance conductivity, increase ECSA, and optimize the adsorption/desorption energy of reaction intermediates through interfacial electronic effects (Figure 12g–i) [112, 133, 134, 140, 178, 179, 180, 181, 182, 183, 184]. However, the performance gain cannot be directly equated with newly created Ce active sites. In CeO_2/_Ni(OH)_2_/carbon foam, the Ni oxidation peak shifts from 1.46 to 1.41 V, and the integrated peak area increases by approximately 1.7‐fold, supporting the role of CeO_2_ in promoting the formation of high‐valent Ni active species [185]. At the CeO_2_/NiFeCr hydroxide interface, DFT indicates that d‐f coupling promotes charge transfer and improves the reaction thermodynamics of host sites [160]. These results support regulation of the host catalyst by CeO_2_, but they cannot quantify the respective contributions of newly created Ce sites versus host regulation, nor can they rule out Ce‐related interfacial activity in other systems. Distinguishing these two contributions should match loading and testing conditions, compare the host catalyst, CeO_2_, physical mixtures, and heterostructures, and combine reliable site normalization with operando spectroscopy; an increase in oxidation peak area or ECSA is not equivalent to a Ce‐site contribution. For example, in carbon material composite strategies, the CeO_2_‐hydroxylated graphene hybrid catalyst (C‐hG) exhibits an OER onset potential superior to IrO_2_. DFT calculations reveal that the covalent interface between surface oxygen atoms of CeO_2_ and low‐hydroxylation‐coverage graphene is key to activating catalytic sites [181]. Single‐atom catalyst (SAC) design further improves atom utilization, such as single atoms of W, Cu, and Co oxides anchored on CeO_2_‐rGO (rGO‐ED‐CeO_2_‐WCuCo‐30). High‐angle annular dark‐field scanning transmission electron microscopy (HAADF‐STEM) and XPS confirm the presence of isolated metal atoms, abundant O_v_s, and multivalent metals [186]. Morphology control and defect engineering are likewise decisive. CoCe/C HFs with three‐dimensional nanoflower‐like structures or nanoneedle arrays are a representative case that renders a substantially larger population of active sites accessible [133]. In ZIF‐67‐derived Co_3_O_4_/CeO_2_ heterostructures, porous channels emerge via the self‐assembly of two‐dimensional nanosheets, an arrangement that facilitates electrolyte diffusion [134]. Additionally, self‐supporting electrode architectures (e.g., directly grown on nickel foam) do away with binders altogether, which, in turn, strengthens mechanical stability and durability under high current densities [135]. A self‐supporting Co/CeO_2_ heterojunction electrode achieves an OER overpotential of 400.3 mV at a high current density of 1 A cm^−2^, and overall water splitting requires a cell voltage of only 1.92 V to reach 1 A cm^−2^ [135]. Representative performance data are summarized in Table 3. In summary, doping, heterostructure construction, defect engineering, and morphology control of CeO_2_‐based electrocatalysts can significantly enhance their catalytic activity, stability, and reaction kinetics in alkaline OER. Whether the lattice oxygen of ceria itself participates directly in the OER cycle, or acts indirectly by modulating the lattice‐oxygen activity of the host, is examined in detail in Section 3.4.

**FIGURE 12:**
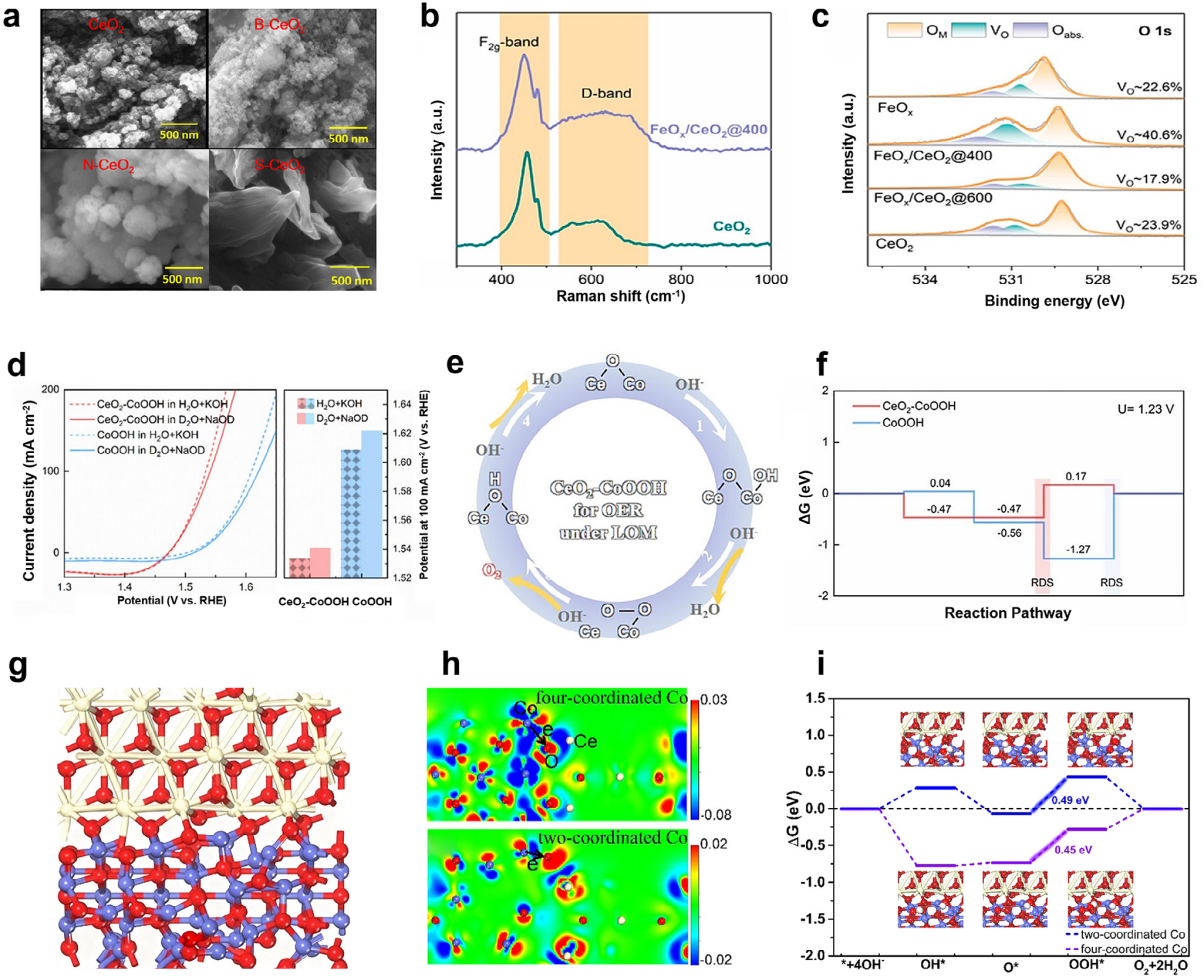
(a) Effect of different non‐metal heteroatom doping on the morphology of CeO_2_. Reproduced with permission [131]. Copyright 2021 Elsevier. (b,c) Abundant O_v_s formed by transition metal doping: (b) Raman spectra of FeO_x_/CeO_2_@400 and CeO_2_; (c) O 1s spectra for CeO_2_, FeO_x_/CeO_2_@400, FeO_x_/CeO_2_@600, and FeO_x_, with the proportions of O_v_ remarked in the panel. Reproduced with permission [132]. Copyright 2023 American Chemical Society. (d–f) Alteration of the reaction pathway by transition metal doping: (d) Polarization curves and corresponding potential comparison of CeO_2_‐CoOOH/NF and CoOOH/NF in KOH and deuterium isotope solution; (e) Schematic illustration of the OER pathway under LOM; (f) OER Gibbs free energy results of CeO_2_‐CoOOH and CoOOH models, respectively. Reproduced with permission [139]. Copyright 2025 John Wiley & Sons. (g,h) Formation of highly active sites via electron transfer at the heterointerface: (g) Relaxed geometric structure of the CeO_2_‐CoO system. (h) Charge density difference maps for the four‐coordinate and two‐coordinate cobalt sites. (i) Free‐energy profiles of the four elementary steps of the OER on the four‐coordinate and two‐coordinate Co sites over the CeO_2_‐CoO surface, along with the optimized adsorption configurations of *OH, *O, and *OOH intermediates. Color code: red O; blue, Co; yellow, Ce; white, H. Reproduced with permission [140]. Copyright 2021 American Chemical Society.

**TABLE 3:** Summary of the performance of CeO_2_‐based electrocatalysts in alkaline electrolytes.

Materials	η@10 mA cm^−2^ (mV)	Tafel slope (mV dec^−1^)	Stability (h)	Electrolyte	Key character affecting the activity	Ref.
CeO_2_/Co_4_N	318	80	10 h @10 mA cm^−2^	1.0 M KOH	In situ CoOOH active sites with O_v_ in CeO_2_ boost OER.	[174]
AuNPs‐decorated hydrothermal CeO_2_ / Ni foam	310	30.25	50 h @ 10 mA cm^−2^	1.0 M KOH	3D CeO_2_ with AuNPs boosting active sites.	[175]
S‑CeO_2_	190	83	20 h @ 10 mA cm^−2^	1.0 M KOH	S doping enriches Vo, leverages Ce redox, builds petal architecture.	[131]
Ni+Fe‐CeO_2_	380	65	20 h @ 10 mA cm^−2^	1.0 M KOH	Mesoporous CeO_2_ with enriched O_v_ and Ni/Fe synergy.	[94]
FeO_x_/CeO_2_	252	45	48 h@ 10 mA cm^−2^	1.0 M KOH	CeO_2_ host supplies Ce^3+^/Ce^4+^ redox and O_v_.	[132]
Ce_0.8_Ni_0.15_Fe_0.05_O_x_	325	65	>1000 cycles CV	1.0 M KOH	Ceria‑confined Ni/Fe: suppressed leaching, stable and active.	[177]
CeO_2_‐CoOOH/NF	249	44.1	72 h @ 100 mA cm^−2^	1.0 M KOH	CeO_2_ induces Ce‐O‐Co coupling boosting LOM and deprotonation.	[139]
CeO_2_‐CoO	296	76.78	130 h @1.55V	1.0 M KOH	CeO_2_‐CoO interface creates O_v_ and low‐coordinate Co sites.	[140]
NiO@CeO_2_	287	156	24 h@1.56V	1.0 M KOH	CeO_2_ supplies redox‐active Ce^3+^/Ce^4+^ that stabilizes Ni sites and speeds OER.	[178]
CoP/CeO_2_	257	53	30 h@ 50 mA cm^−2^	1.0 M KOH	Interfacial CoP/CeO_2_ coupling creates electron transfer / interface sites and CeO_2_ supplies oxygen‐vacancy‐rich redox.	[179]
RuO_2_/CeO_2_	350	74	12 h @ 1.6 V	1.0 M KOH	Defective, high‐surface‐area CeO_2_ support stabilizes RuO_2_, promotes charge transfer and electrolyte access.	[180]
CeO_2_ NCs anchored on hydroxylated GO	440	60	0.70→1.63 V by 2000 cycles	0.1 M KOH	Hydroxyl‐driven covalent Ce‐O‐C activation at the ceria‐graphene interface.	[181]
g‐C_3_N_4_/ CeO_2_ / Fe_3_O_4_	400	74	14 h @ 10 mA cm^−2^	1.0 M KOH	Reducible CeO_2_ aids tuning electronic states for enhanced activity.	[182]
Ni_0.8_Co_0.2_Ce_0.1_/NF	207	39.7	36 h @ 100 mA cm^−2^	1.0 M KOH	Ce^3+^/Ce^4+^ redox and O_v_ boost charge transfer and stabilize active NiCo oxyhydroxide.	[183]
Co_3_O_4_/CeO_2_/C	274	82.6	12 h @ 10 mA cm^−2^	1.0 M KOH	CeO_2_ introduces O_v_ and tunes Co_3_O_4_ electronic structure.	[134]
CeO_2_@PANI	226	36	/	1.0 M KOH	CeO_2_ disperses over PANI supplying Ce^3+^/Ce^4+^ redox sites, raising conductivity and active‐site density.	[184]
rGO‐ED‐CeO_2_‐WCuCo	283	57.03	24 h @ 10 mA cm^−2^	1.0 M KOH	CeO_2_ Ce^3+^/Ce^4+^ redox and O_v_ tune OER intermediate adsorption.	[186]
Co/CeO_2_@CF	214	79.3	70 h @ at 500 mA cm^−2^	1.0 M KOH	CeO_2_ tunes interfacial charge redistribution and O_v_ to optimize OER intermediate adsorption.	[135]

### 4.2 Acidic Electrolytes

The OER in an acidic environment is a core process in proton exchange membrane (PEM) water electrolysis technology, an emerging and more efficient method for water splitting. However, the highly corrosive nature of acidic media poses significant challenges for catalyst development [187, 188, 189, 190]. In recent years, CeO_2_‐based composite materials have exhibited a significant enhancement in stability for the acidic OER through catalyst structure engineering strategies, such as interface engineering and valence state regulation. In terms of interface engineering, CeO_2_ compounded with a main catalyst in the form of a heterojunction (e.g., CeO_2_/Co_3_O_4_) enables OER at low overpotentials in acidic environments and further suppresses acid corrosion and enhances stability through carbon coating that promotes OH coverage (Figure 13a) [191]. Similarly, composites of CeO_2_ with iridium oxides (IrO_x_), such as IrO_x_/CeO_2_ nanowires, utilize the electron buffering effect of CeO_2_ to modulate Ir valence states, achieving an overpotential of 220 mV and 300 h of stability in acidic media, with a mass activity as high as 167 A g^−1^ [143]. The introduction of CeO_2_ enhances OER performance primarily through the following mechanisms: First, the O_v_ and redox properties of CeO_2_ promote electron transfer and intermediate adsorption. As shown in Figure 13b, in CeO_2_/Co‐Ni‐P‐O_x_, CeO_2_ forms Ce‐O‐Co bonds with cobalt oxide, increases the valence state of Co, and in situ generates Co^3+^‐O active sites, lowering the deprotonation energy barrier of water molecules. DFT‐based analysis identifies the reducible Co^3+^‐O sites as the origin of the optimized OH and OOH adsorption [192]. Equally critical is the interfacial interaction between CeO_2_ and noble metals (e.g., Ir, Ru): strong metal‐support interactions (MSI) suppress metal dissolution, thereby markedly enhancing stability (Figure 13c,e–g) [144]. The effect is plainly seen in the Ir dissolution rate of IrO_x_/CeO_2_, which is a mere 0.81%, a sharp contrast to the 12.35% recorded for carbon‐supported analogues [145]. Furthermore, morphology and the adopted composite strategy further dictate performance. Zero‐dimensional/two‐dimensional (0D/2D) heterostructures, such as CeO_2_ quantum dots decorating SrIrO_3_ nanosheets (Figure 13d), expand the population of interfacial active sites; carbon supports (CeO_2_@C), on the other hand, confer improved conductivity and dispersibility. Table 4 summarizes representative acidic OER performance data. It should be noted, however, that an excessive CeO_2_ content can dilute the proportion of active sites and, in turn, compromise catalytic activity [141, 142]. In summary, CeO_2_‐based electrocatalysts provide efficient and stable solutions for acidic OER through valence state regulation, interface engineering, and electron buffering effects. Future work needs to further explore low‐cost, large‐scale preparation and practical PEMWE applications.

**FIGURE 13:**
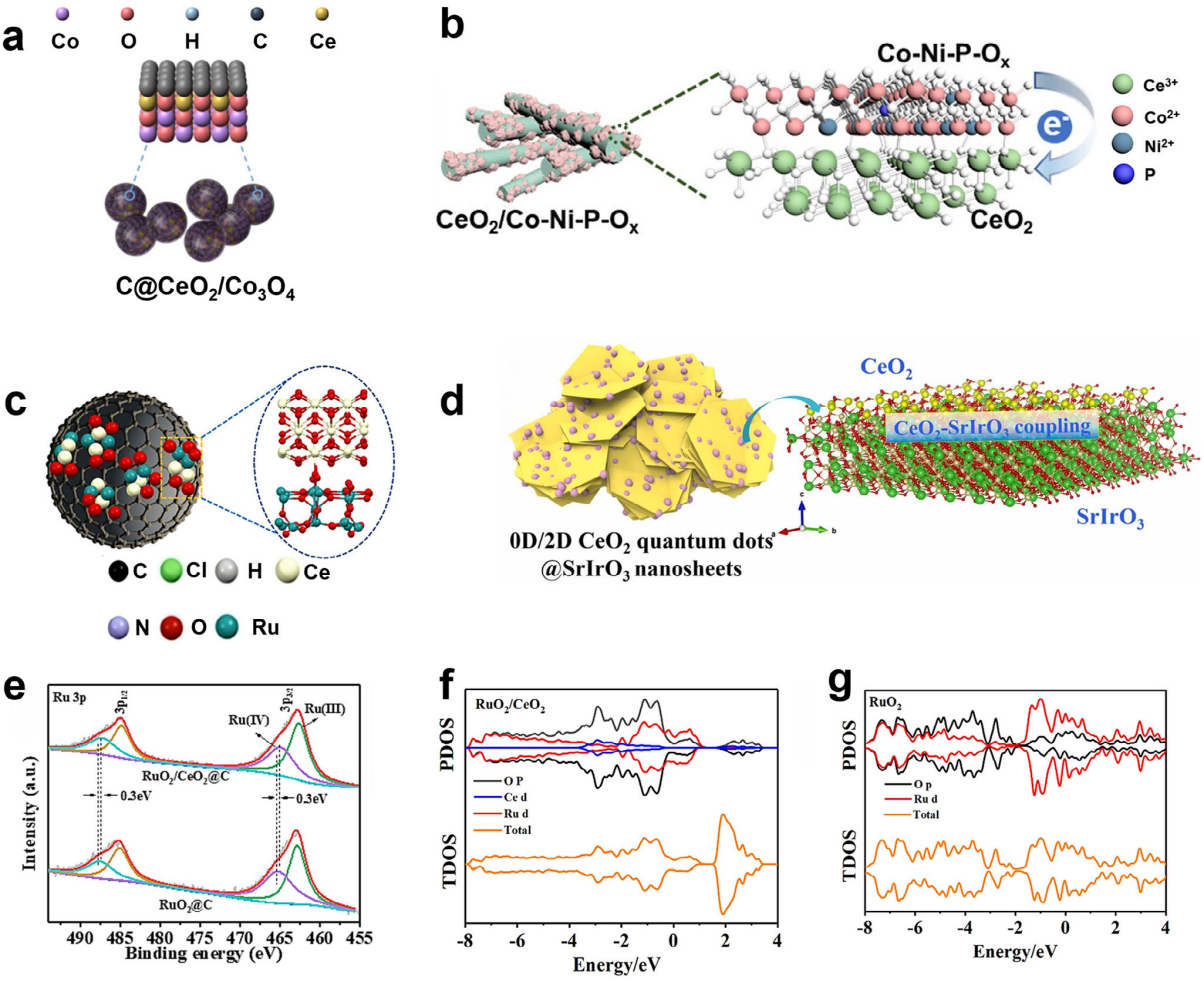
(a–d) illustrates different composite strategies of CeO_2_‐based composite materials to enhance their OER performance in acidic solutions: (a) Schematic diagram of the mechanism for C@CeO_2_/Co_3_O_4_. Reproduced with permission [191]. Copyright 2023 American Chemical Society; (b) Schematic diagram of the mechanism for CeO_2_/Co‐Ni‐P‐O_x_. Reproduced with permission [192]. Copyright 2023 American Chemical Society; (c) Schematic diagram of the mechanism for RuO_2_/CeO_2_@C. Reproduced with permission [144]. Copyright 2023 Elsevier; (d) Schematic diagram of the mechanism for CeO_2_@SrIrO_3_. Reproduced with permission [142]. Copyright 2022 Elsevier. (e–g) Modifying the valence state of the main catalyst through electron regulation to reduce its leaching. (e) Ru 3p XPS spectra of RuO_2_/CeO_2_@C and RuO_2_@C; (f) The projected density of states for RuO_2_/CeO_2_; (g) The projected density of states for RuO_2_. Reproduced with permission [144]. Copyright 2023 Elsevier.

**TABLE 4:** Summary of the performance of CeO_2_‐based electrocatalysts in acidic electrolytes.

Materials	η@10 mA cm^−2^ (mV)	Tafel slope (mV dec^−1^)	Stability (h)	Electrolyte	Key character affecting the activity	Ref.
C@CeO_2_/Co_3_O_4_	425	98.5	50 h @10 mA cm^−2^	0.5 M H_2_SO_4_	CeO_2_ modulates Co valence and promotes *OH coverage, boosting acidic OER.	[191]
IrO_x_/CeO_2_	220	63	300 h @ 10 mA cm^−2^	0.5 M H_2_SO_4_	CeO_2_ acts as an electron buffer that stabilizes Ir oxidation states and promotes OER activity.	[143]
CeO_2_/Co_2_NiP_0.03_O_x_	262	32.9	5 h @ 5 mA cm^−2^	0.5 M H_2_SO_4_	CeO_2_ heterojunction creates stable active Co^3+^‐O sites boosting OER activity.	[192]
RuO_2_/CeO_2_@C	170	56.6	100 h @50 mA cm^−2^	0.5 M H_2_SO_4_	RuO_2_/CeO_2_ heterointerface with CeO_2_ tunes Ru electronic structure, boosts OER.	[144]
IrOx/CeO_2_	263.4	43.04	10 h @ 10 mA cm^−2^	0.1 M HClO_4_	O_v_ in CeO_2_ induces active Ir^3+^ boosting OER.	[145]
4CeO_2_@SrIrO_3_	238	71.7	50 h @ 10 mA cm^−2^	0.5 M H_2_SO_4_	CeO_2_ quantum‑dot decoration stabilizes SrIrO_3_ and promotes OER activity.	[142]
RuO_2_−CeO_2_−CC	180	58.9	1000 h @ 10 mA cm^−2^	0.5 M H_2_SO_4_	Ru‐O‐Ce bridging and Ce‐induced lattice strain lower the OER barrier while suppressing Ru dissolution.	[99]
Ce–IrO_2_	194	^—^	400 h @ cell 1.8 V	0.5 M H_2_SO_4_	CeO_2_‐induced compressive strain switches OER to OPM.	[100]
Ir‐CeO_2_ ‐C	283	67.7	10 h @ 10 mA cm^−2^	0.5 M H_2_SO_4_	Interfacial Ir‐O‐Ce bonding modulates Ir electron density to create high‐activity OER sites.	[193]

### 4.3 Seawater Electrolytes

CeO_2_‐based electrocatalysts exhibit outstanding OER activity and stability in seawater electrolysis. Their mechanisms of action primarily include Cl^−^ blocking, electronic structure modulation, surface reconstruction optimization, and other aspects [194, 195]. Based on the Hard and Soft Acids and Bases principle (HSAB), CeO_2_, as a hard Lewis acid, preferentially adsorbs OH^−^ over Cl^−^. This creates an OH^−^‐enriched local microenvironment on the electrode surface, effectively inhibiting Cl^−^ adsorption and the consequent competitive chlorine evolution reaction (ClER) and electrode corrosion processes (Figure 14a–c) [196, 197]. For instance, introducing CeO_2_ and Cl^−^ intercalation into CoFe LDH (CoFeOOH‐Cl^−^/CeO_2_) results in a catalyst that, in alkaline seawater, shows an overpotential of only 329 mV at a current density of 1000 mA cm^−2^ and can operate stably for 1000 h. As shown in Figure 14d, the mechanism originates from the Lewis acidity of CeO_2_, which preferentially binds OH^−^, while intercalated Cl^−^ lowers the adsorption energy of chloride on the metal centers. This dual effect that substantially enhances reaction selectivity and structural robustness [198]. Beyond modulating adsorption selectivity, CeO_2_ also establishes a physical electrostatic barrier against Cl^−^ erosion: its point of zero charge (PZC) is roughly 7, so under alkaline conditions the surface acquires a negative charge and repels the like‐charged Cl^−^ ions, thereby suppressing electrode corrosion and side reactions [146]. Through interface engineering, ceria further safeguards the active phase by combining physical isolation with electronic modulation. The resulting in situ architecture defends the catalyst against corrosion while preserving high OER selectivity [146, 199]. In situ characterization, for instance, tracks the dynamic evolution of CeO_2_ during OER and confirms its conversion into a nanoparticle film (ca. 1 nm thick) that coats the active phase surface, thereby boosting stability (Figure 14e) [146]. It is worth noting that this CeO_2_ overlayer typically possesses a disordered structure that allows OH^−^ ions to diffuse unhindered to the active sites beneath, ensuring sustained OER activity. An electrode prepared by a two‐step electrodeposition method, Ce‐Co(OH)_2_@FeOOH, utilizes this mechanism, and operates stably for 2500 h at a high current density of 2 A cm^−2^ in simulated seawater without hypochlorite formation [146]. On the other hand, the introduction of CeO_2_ optimizes the electronic structure of the catalyst. Its unique adjustable O_v_ and Ce^3+^/Ce^4+^ redox pairs can modulate the valence state and electron configuration of adjacent metal active centers (e.g., Co, Ni, Fe) through strong electronic interactions, thereby lowering the OER energy barrier and promoting the in situ formation of highly active species (e.g., CoOOH) [200, 201]. In the CeO_2_/CoNiP heterojunction, the negative zeta potential of CeO_2_ under alkaline conditions helps electrostatically repel Cl^−^, reducing the leaching of the active component cobalt (Figure 14f). Simultaneously, CeO_2_ promotes the adsorption of the *OH intermediate through its O_v_, significantly reducing the energy barrier of the rate‐determining step [197]. In the NiFe‐LDH system, the synergistic effect of CeO_2_ and (W_2_O_7_) [2−] intercalation, by expanding the interlayer spacing and enhancing Lewis acidity, causes Ni and Fe sites to exhibit higher valence states, preferentially adsorbing OH^−^. DFT calculations confirm that the Cl^−^ adsorption energy weakens from −0.52 to −0.25 eV, while the OH^−^ adsorption energy strengthens from −0.24 to −0.69 eV (Figure 14 g) [200]. The above adsorption differences not only involve chemical bonding interactions but may also be related to changes in the local coordination environment of Ni/Fe sites. In terms of chemical bonding, surface hydroxyl groups on CeO_2_ can coordinate with Ce sites through the oxygen end to form Ce‐OH, providing a basis for the specific adsorption of oxygen‐containing species [202]. In the aforementioned example where CeO_2_ and (W_2_O_7_) [2−] intercalation jointly regulate the electronic structure of Ni/Fe sites, from the perspective of ligand field theory, changes in ligand arrangement, local symmetry, and bond length may further tune the d‐orbital energy levels of Ni/Fe sites and metal 3d‐O‐2p orbital hybridization, thereby affecting the adsorption behavior of oxygen‐containing intermediates [203, 204]. For example, in the La‐doped NiFe alloy, the introduction of La upshifts the d‐band center of NiFe from −1.15 to −0.99 eV (NiFeMo: −1.03 eV), thereby enhancing OOH adsorption and lowering the RDS (O → *OOH) energy barrier [203]. Moreover, previous studies have shown that the introduction of Ce can modulate the electronic structure and metal‐oxygen covalency of neighboring metal centers through Ce 4f‐O 2p‐transition metal 3d orbital coupling. For example, in Ce single‐atom‐modified CoO, the introduction of Ce negatively shifts the Co 2p_3/2_ binding energy by about 0.50 eV and shortens the Co‐O bond length from about 1.90 to 1.77 Å, thereby enhancing Co‐O covalency and optimizing the adsorption of oxygen‐containing intermediates [78], offering a possible orbital‐level explanation for enhanced OH^−^ adsorption. The preferentially adsorbed OH^−^ may also limit Cl^−^ adsorption through competitive site occupation. The microscopic morphology and structural design of the catalyst are also crucial. A bionically designed CeO_2_/Ni/carbon cloth catalyst mimicking the sea anemone tentacle structure provides a high specific surface area and a superhydrophilic surface, achieving an overpotential of 220 mV at 10 mA cm^−2^ in 1 M KOH + 0.6 M NaCl. CeO_2_ induces a blue shift in the d‐band center of Ni, optimizing the adsorption energy of oxygen intermediates while enhancing Cl^−^ repellency [201]. Table 5 summarizes the reported seawater OER performance of representative CeO_2_‐based catalysts. In summary, CeO_2_‐based OER catalysts show great potential in seawater environments.

**FIGURE 14:**
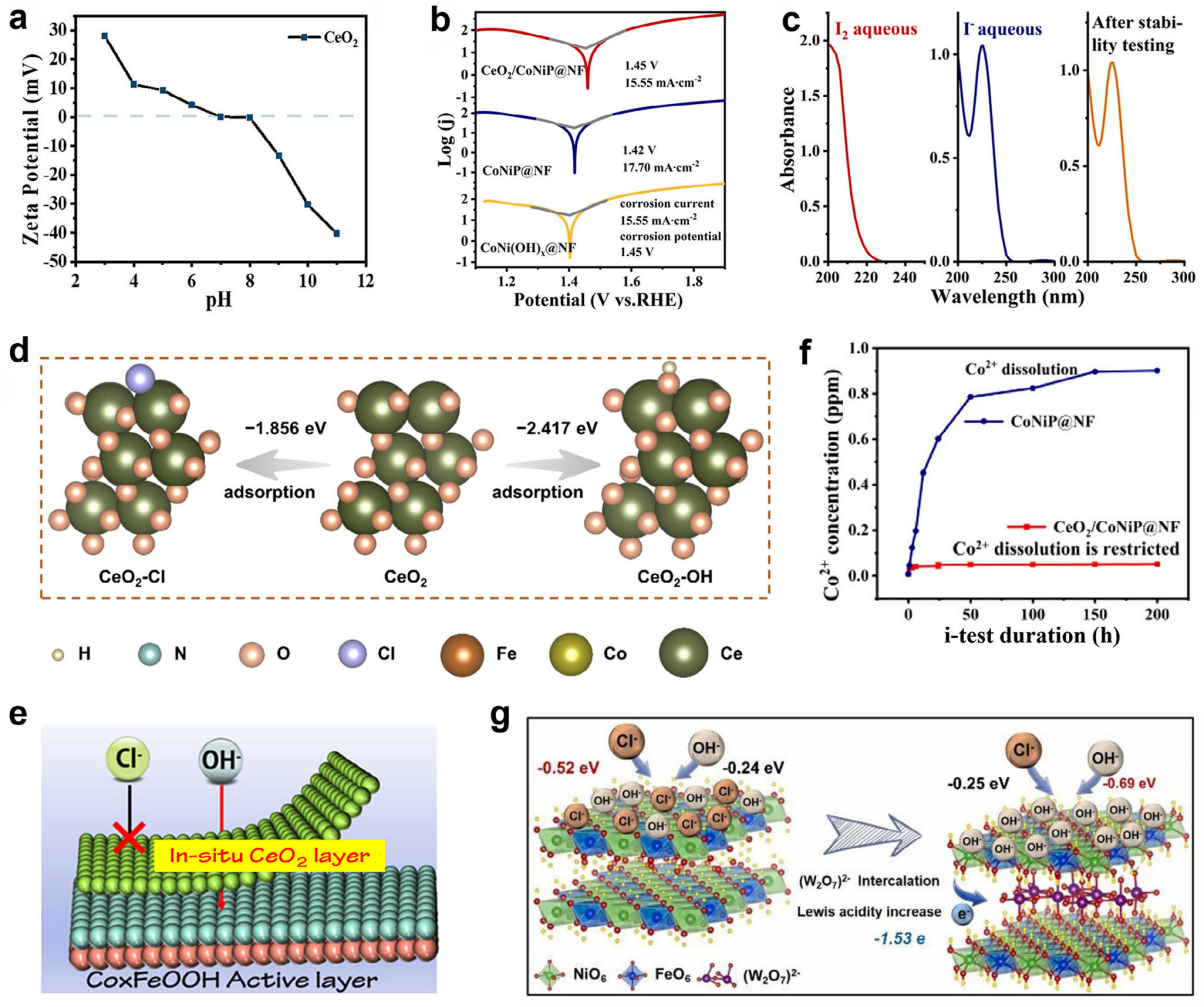
(a–g) Protective mechanisms and adsorption regulation in CeO_2_‐containing OER catalysts for alkaline seawater electrolysis. (a) Zeta potential of CeO_2_ as a function of pH, approaching zero around pH 7–8 and becoming negative at higher pH. (b) Potentiodynamic polarization curves of CeO_2_/CoNiP@NF, CoNiP@NF and CoNi(OH)_x_@NF in simulated alkaline seawater, used to compare corrosion potentials and corrosion current densities. (c) UV–vis spectra of 0.5 M KI, an I_2_ aqueous solution and the electrolyte treated with KI after a 250 h durability test in simulated alkaline seawater; the post‐test sample exhibits an iodide‐like spectrum without a detectable I_2_ signal, consistent with no hypochlorite detected by this assay. Reproduced with permission [197]. Copyright 2025 American Chemical Society. (d) DFT‐calculated adsorption energies of *Cl (−1.856 eV) and *OH (−2.417 eV) on CeO_2_ with the corresponding schematic illustration, showing more favorable OH adsorption in the calculated model, with both adsorption energies being negative. Reproduced with permission [198]. Copyright 2025 Elsevier. (e) Schematic of the proposed protective action of an approximately 1 nm CeO_2_ surface layer generated in situ on the Co_x_FeOOH active layer, suppressing Cl^−^ access while permitting OH^−^ transport to the underlying active sites. Reproduced with permission [146]. Copyright 2025 American Chemical Society. (f) Dissolved Co concentrations measured by ICP‐OES for CeO_2_/CoNiP@NF and CoNiP@NF during a 200 h test at 500 mA cm^−2^ in simulated alkaline seawater, showing suppressed Co leaching after CeO_2_ modification. Reproduced with permission [197]. Copyright 2025 American Chemical Society. (g) Schematic illustration of the HSAB principle and adsorption regulation on NiFeOOH before and after (W_2_O_7_) [2−] intercalation in the CeO_2_‐modified NiFe‐LDH system: the calculated adsorption energy changes from −0.52 to −0.25 eV for Cl^−^ and from −0.24 to −0.69 eV for OH^−^, indicating weaker Cl^−^ adsorption and stronger OH^−^ adsorption following intercalation. Reproduced with permission [200]. Copyright 2023 Elsevier.

**TABLE 5:** Summary of the performance of CeO_2_‐based electrocatalysts in seawater electrolytes.

Materials	η@10 mA cm^−2^ (mV)	Tafel slope (mV dec^−1^)	Stability (h)	Electrolyte	Key character affecting the activity	Ref.
0.25CeO_2_/NiCoP	233	^—^	140 h @ 110 mA cm^−2^ (two‐electrode overall water splitting)	1.0 M KOH + 0.5 M NaCl (S)	CeO_2_ incorporation resists Cl^−^ corrosion for enhanced seawater OER stability.	[194]
CeO_2_/D‐NiFe‐LDH@Cu_(w)_	230 mV @100 mA cm^−2^	72.6	80 h @ 400 mA cm^−2^	1 M KOH + natural seawater (N)	CeO_2_ provides Ce‐O‐Ni sites that raise Ni Lewis acidity, boosting OH^−^ affinity over Cl^−^ in seawater.	[195]
CeO_2_‐Co_2‐x_Ni_x_P@C	323	84.6	20 h @ 1.6 V	1 M KOH + seawater (N)	CeO_2_ suppresses competitive ClOR preserving selective OER activity in seawater.	[196]
CeO_2_/CoNiP@NF	249	66	200 h @ 500 mA cm^−2^	1.0 M KOH + NaCl (simulated seawater, [Cl^−^] ≈ 0.5 M) (S)	The negative ζ potential of CeO_2_ electrostatically repels Cl^−^ and suppresses Co leaching, stabilizing OER in seawater.	[197]
CoFeOOH‐Cl^−^/CeO_2_	329 mV @ 1000 mA cm^−2^	64.58	1000 h @ 1000 mA cm^−2^	1 M KOH + seawater (N)	Interlayer Cl^−^ repels aggressive Cl^−^while hard‐Lewis‐acidic CeO_2_ enriches OH^−^ to protect and activate CoFeOOH in seawater.	[198]
CeO_x_@NiCo_2_O_4_/NF	238	80	60 h @ 10 mA cm^−2^	0.1 M KOH (No attenuation in seawater) (S)	CeO_x_‐modified NiCo_2_O_4_ interface engineering stabilizes bifunctional seawater electrolysis.	[199]
NiFe LDH‐CeW@NFF	386.7	81.2	100 h @ 1000 mA cm^−2^	1 M KOH + 0.5 M NaCl (S)	CeO_2_ with O_v_ optimizes OER intermediate adsorption, synergy with (W_2_O_7_) [2−] delivers OH^−^‐over‐Cl^−^ selectivity in seawater.	[200]
CeO_2_/Ni/CC	220	104	100 h @ 10 mA cm^−2^	0.6 M NaCl + 1.0 M KOH (S)	CeO_2_ doping suppresses Cl^−^ adsorption and corrosion while optimizing OER in seawater.	[201]
Fe‐Ni_2_P/CeO_2_/NF	256 mV @200 mA cm^−2^	112.7	500 h @ 600 mA cm^−2^	1 M KOH + 0.5 M NaCl (S)	CeO_2_ enriches Fe‐Ni_2_P electrons, weakens *Cl, boosts seawater OER.	[205]

Table note: S = simulated alkaline seawater, prepared by adding NaCl (typically 0.5–0.6 M) to KOH; it reproduces the Cl^−^ concentration of seawater but not its Mg^2+^, Ca^2+^, SO_4_2^−^, Br^−^, dissolved organic matter or microorganisms. N = natural seawater, filtered and alkalized with KOH. Because data obtained in simulated seawater do not capture the precipitation of Mg(OH)_2_, Ca(OH)_2_ and CaCO_3_, impurity poisoning or biofouling, values measured under the two conditions should not be compared directly. Overpotentials are given at the current density indicated in parentheses.

## 5 Conclusions and Perspectives

As shown in Figure 15, this review systematically summarizes recent research progress on CeO_2_‐based electrocatalysts for OER. With the rapid advancement of operando characterization techniques and the growing understanding of dynamic catalytic processes, increasing evidence demonstrates that the functionality of CeO_2_ extends far beyond static interfacial regulation. Originating from the highly localized yet electronically accessible Ce 4f orbitals, the reversible Ce^3+^/Ce^4+^ redox cycle of CeO_2_ enables dynamic electron storage and transfer coupled with O_v_s evolution. These intrinsic properties enable CeO_2_ to mediate catalytic evolution across multiple length scales, ranging from atomic‐level electronic structure modulation to macroscopic structural stabilization. Through multiscale structural engineering, from hierarchical porous architectures to atomically dispersed configurations, and advanced synthetic strategies including hydrothermal growth, electrodeposition, and dealloying, CeO_2_ has been integrated with a broad range of non‐precious‐metal compounds (e.g., Co‐, Ni‐, and Fe‐based materials) and noble‐metal oxides (e.g., IrO_2_ and RuO_2_), constructing intimate interfaces with tailored gradient d‐p‐f electronic coupling pathways. At these interfaces, CeO_2_ regulates OER activity and durability through a synergistic “redox–lattice oxygen interplay,” involving dynamic charge redistribution, oxygen vacancy‐mediated lattice oxygen activation, and adaptive surface reconstruction:

1. First, the plentiful O_v_ serve dual functions: they act as active centers for the preferential adsorption and activation of water molecules or hydroxyl groups, and they induce interfacial charge redistribution, which optimizes the adsorption free energies of the OER intermediates (^*^OH, ^*^O, ^*^OOH). This may even alter the reaction pathway from the conventional AEM to the more efficient LOM.
2. Second, the strong electronic interaction between CeO_2_ and the primary catalyst can effectively modulate the d‐band center of the active metal, optimize its electronic structure and lower the reaction energy barrier.
3. CeO_2_ both facilitates and stabilizes the dynamic surface reconstruction of the primary catalyst. The O_v_‑rich interface preferentially adsorbs OH^−^. This adsorption supplies the driving force for in situ hydroxylation/oxidation of transition metal compounds (sulfides, phosphides, etc.), thereby accelerating the generation of highly active amorphous (oxy)hydroxide phases. The ongoing Ce^3+^/Ce^4+^ redox cycle, together with O_v_, sustains the long‑term stability of the reconstructed interface.

**FIGURE 15:**
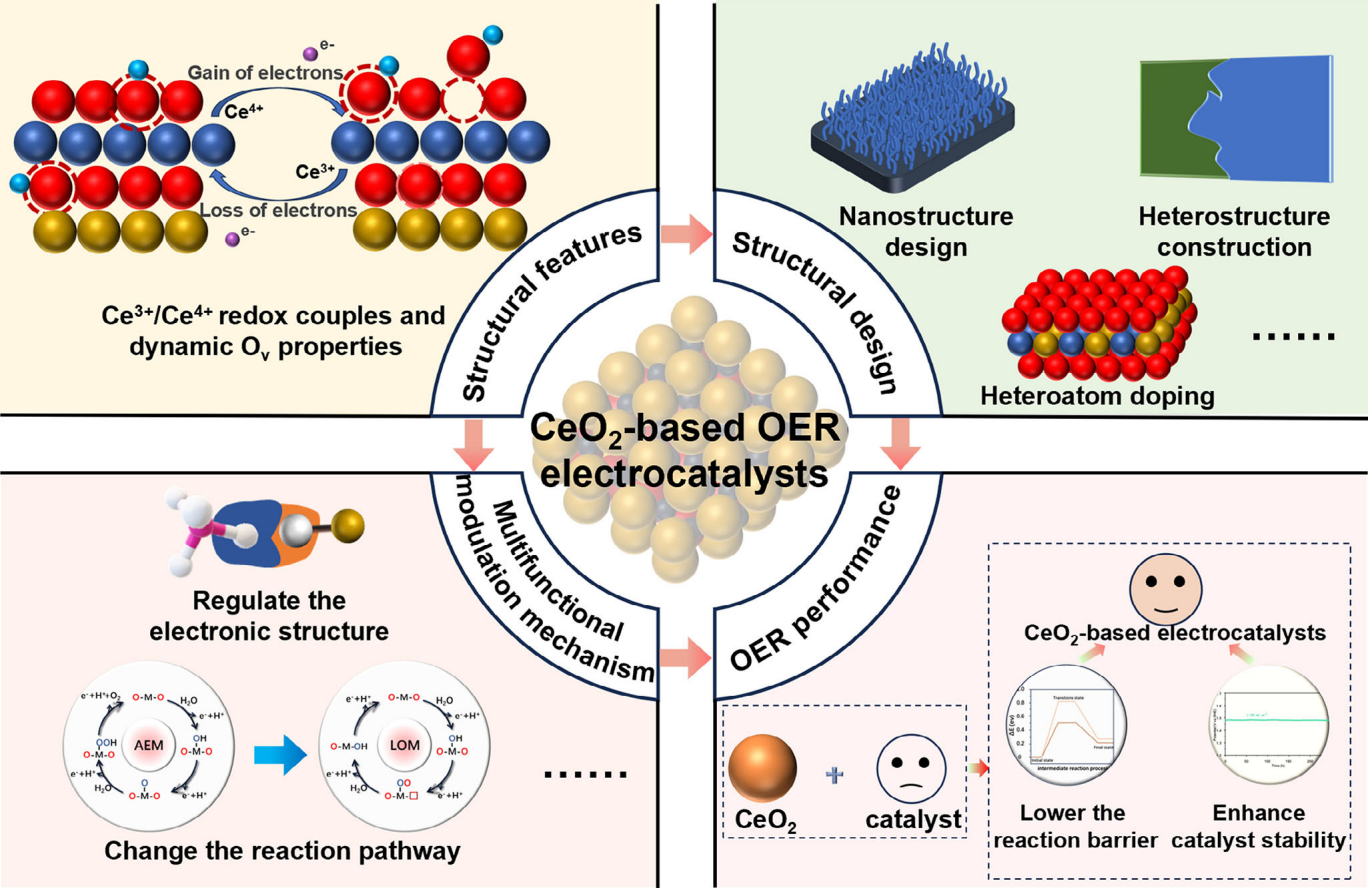
Ce redox and multiscale engineering in CeO_2_‐based OER electrocatalysts.

It is worth noting that recent studies have further indicated that such multi‐level synergy endows CeO_2_‐based electrocatalysts with striking adaptability across distinct electrolytic environments. In aggressively acidic media, CeO_2_ operates as an “electron buffer layer” that pins the valence state of precious metals and suppresses their leaching, leading to a pronounced enhancement in durability. Under alkaline conditions, combining CeO_2_ with varied non‐noble metal components allows the catalyst to fully exploit its O_v_ and Ce^3+^/Ce^4+^ redox couple, thereby attaining high activity alongside stability. When deployed in highly corrosive seawater electrolytes, HSAB theory predicts that the CeO_2_ surface, behaving as a hard Lewis acid, preferentially binds OH^−^ while electrostatically repelling Cl^−^. This creates a localized alkaline microenvironment at the electrode surface, fundamentally inhibiting the ClER and electrode corrosion, fully demonstrating its selective protection capability.

Leveraging its unique oxygen‐vacancy dynamics, interfacial electronic modulation, and capacity to trigger dynamic reconstruction, CeO_2_ effectively combines the roles of a structural promoter for boosting stability and a functional promoter for optimizing the electronic structure and reaction pathway. This lays a robust material groundwork and provides theoretical direction for designing more efficient, durable, and cost‑competitive next‑generation OER electrocatalytic systems than traditional precious‐metal systems, especially for large‑scale hydrogen production via water electrolysis.

Despite significant achievements in the research of CeO_2_‐based OER electrocatalysts, their industrial application still faces numerous challenges. Future research should delve deeper into the following directions (Figure 16):

1. First, the static understanding of the action mechanisms of CeO_2_ must be broken, moving toward in‐depth dynamic mechanism analysis under operating conditions. The current understanding of the cocatalytic function of CeO_2_, such as how it promotes lattice oxygen participation to modulate reaction pathways, facilitates interfacial charge transfer, and stabilizes low‐valence active centers, still relies heavily on idealized models constructed by ex situ characterization and density functional theory calculations. However, under real oxygen evolution potentials and in highly alkaline or acidic corrosive media, CeO_2_ itself and the interface between CeO_2_ and the primary catalyst are far from static. Future work needs to integrate multiple in situ characterization techniques. Although there have been in situ studies based on Ce oxidation state changes and oxygen vacancy regulation, the issues related to “dynamic evolution of the working state” are still in the exploration stage. In particular, whether the lattice oxygen of CeO_2_ itself directly enters the OER catalytic cycle or CeO_2_ mainly regulates the lattice‐oxygen activity of neighboring active phases remains unresolved; selective ^18^O isotope labeling coupled with DEMS/OLEMS is therefore required to distinguish these possibilities. Further monitoring and analysis through the following in situ characterization methods are required. For instance, in situ XAFS can quantitatively analyze the continuous fluctuation of the Ce^3+^/Ce^4+^ ratio under applied potential, as well as the dynamic evolution of oxygen vacancy concentration and coordination unsaturation. Operando Raman/XRD and EPR can provide complementary information on active‐phase reconstruction and defect evolution. In situ environmental transmission electron microscopy (ETEM) and electrochemical liquid‐phase TEM (ELP‐TEM) can capture, at the nanometer or even atomic scale, the dynamic reconstruction of the interface between CeO_2_ nanoislands and active oxyhydroxide, the formation of an amorphous layer, particle migration and agglomeration, and the dissolution–redeposition cycle of components. Through these and other in situ characterizations, future work may precisely reveal the evolution laws of the true active centers and the critical conditions for reaction pathway switching.
2. Second, attention should be paid to designing interface structures with “self‐healing” capabilities. Under high current density and strong polarization, the surface oxidative reconstruction, cation dissolution, and lattice oxygen consumption lead to irreversible deactivation of the catalyst. Extreme environments such as acidic media and seawater make the durability bottleneck even more critical. Coupling the unique reversible Ce^3+^/Ce^4+^ redox, oxygen storage/release capacity, and adaptive lattice reconstruction of CeO_2_ provides a chemical driving force for a self‐healing interface. Existing studies have shown that CeO_2_ can promote oxygen species exchange between the catalyst and the electrolyte and exhibits a certain “self‐healing” behavior. However, to date, operando evidence for Ce valence cycling has not been widely observed. On this basis, it can be further speculated that its self‐healing process may originate from the dynamic cycle between Ce^3+^/Ce^4+^ and oxygen vacancies: when local oxygen deficiency or coordination unsaturation occurs at the interface during OER, CeO_2_ can buffer the local oxidation state by forming oxygen vacancies and Ce^3+^/Ce^4+^ transformation, while promoting the migration and exchange of oxygen‐containing species such as OH^−^ to defect sites, allowing the damaged metal‐oxygen coordination environment to be recompensated; as the oxygen sites recover, Ce^3+^ is further reoxidized to Ce^4+^, thereby forming a reversible “defect generation—oxygen species compensation—local structure recovery” process. This dynamic process is expected to regulate the interfacial charge distribution and local oxygen chemical potential, reduce structural degradation caused by over‐oxidation, and indirectly inhibit the irreversible dissolution of active metals, thereby maintaining the long‐term stability of the OER active structure. It should be pointed out that this self‐healing is more likely to manifest as dynamic recovery of oxygen defects and local coordination structures, rather than complete regeneration of metal components that have already been extensively dissolved. To translate this proposed self‐healing behavior into durable electrodes, interface design should ensure stable contact between CeO_2_ and the OER active phase while preserving accessible redox sites, strong substrate adhesion, and uniform composition across large areas. Simple, low‐waste, and reproducible synthesis routes should be developed alongside catalyst layers compatible with electrode fabrication and, where applicable, membrane electrode assembly. The amount and distribution of CeO_2_ must also be optimized: its limited electronic conductivity may increase resistance in thick or highly loaded catalyst layers and reduce the fraction of material that participates in OER. For acidic operation, possible Ce dissolution should be quantified; where a proton‐exchange membrane is used, any migration of dissolved Ce species into the membrane or ionomer and its potential effect on ionic transport should be examined. These issues make the transition from laboratory evaluation to industrial operation particularly important. Most CeO_2_‐based OER catalysts are assessed on small electrodes in three‐electrode cells, whereas large‐area electrodes and complete electrolyzers operate at higher current densities for much longer periods. On scale‐up, bubble accumulation, mass‐transport limitations, ohmic losses, and local electrolyte changes may obscure the activity and stability benefits measured in the laboratory. Accordingly, the proposed self‐healing function should be verified under practically relevant current densities, catalyst loadings, temperatures, and electrolyte compositions, including start–stop or fluctuating‐load operation where relevant. Beyond overpotential and Tafel slope, full‐cell voltage, energy efficiency, Faradaic efficiency, gas production, long‐term voltage degradation, Ce and active‐metal dissolution, and post‐operation interfacial changes should be reported. Relating these results to catalyst loading, Ce utilization, and preliminary material and energy costs would clarify whether the mechanistic advantages of CeO_2_ can be retained in scalable electrodes and translated into durable electrolyzer operation.
3. Third, machine learning should be actively fused with high‐throughput automated robotic experimentation platforms to accelerate the rational design and iterative optimization of CeO_2_‐based OER electrocatalysts. Multidimensional operando characterization will generate massive, multimodal dynamic structure–activity data, from which traditional analytical methods can scarcely extract the complex nonlinear relationships buried within. Through machine learning, one can directly learn from the data the deep mapping linking key features, such as Ce^3+^/Ce^4+^ dynamic evolution, oxygen‐vacancy migration, and interfacial restructuring to catalytic activity, selectivity and self‐healing capacity, thereby constructing stability and deactivation prediction models that operate across diverse working conditions. These models, in turn, inversely guide the design of catalyst compositions and interfacial architectures endowed with superior self‐repair ability and corrosion resistance. Concurrently, coupling with automated robotic experimentation platforms enables the efficient synthesis and screening of large candidate libraries following the predictions of the machine‐learning models, forging a closed “prediction‐synthesis‐characterization‐feedback” loop that rapidly validates and refines the models. This integration not only promises to substantially compress the development cycle from mechanistic understanding to industrially relevant process windows, but also reveals multi‐component synergy and dynamic adaptation mechanisms that tend to elude empirical intuition. It thereby furnishes an entirely new research paradigm for breaking through durability bottlenecks in complex industrial scenarios, including acidic electrolytes, high current densities, seawater, and the like.

**FIGURE 16:**
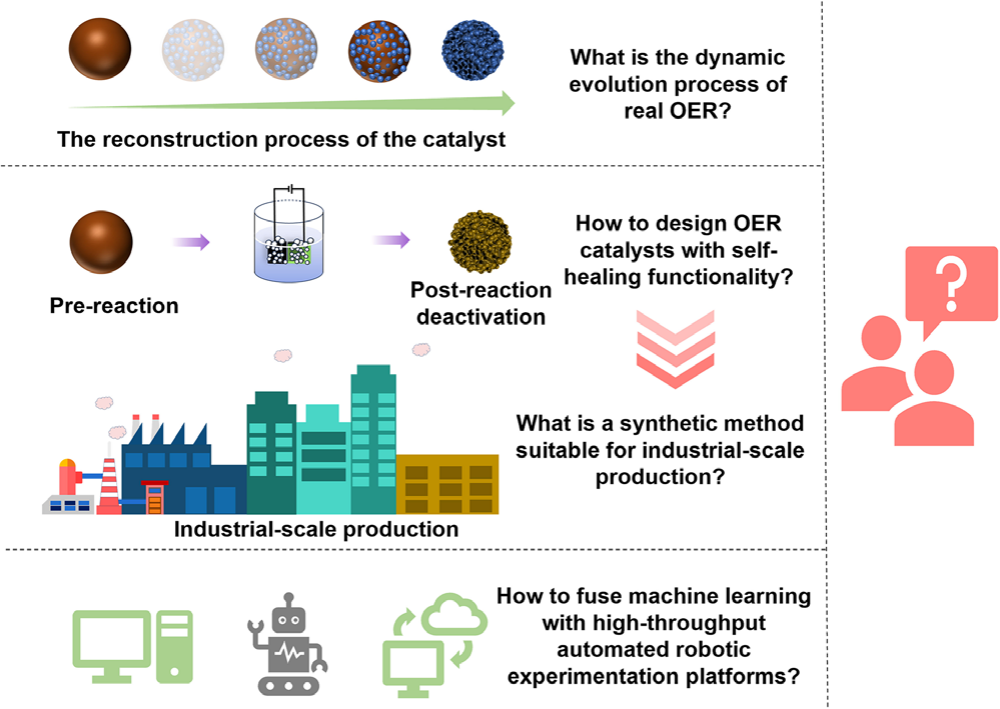
Future perspectives of CeO_2_‐based OER electrocatalysts.

In summary, future breakthroughs require a deeper mechanistic understanding, innovative design concepts, substantial advances in the performance of material systems under industrial scenarios, including acidic environments, high current densities, and tolerance to complex media, as well as the integration of machine learning with automated experimentation to accelerate data‐driven catalyst discovery. This will provide crucial OER material support for the clean energy transition.

## Author Contributions

**Zhen Cui**: writing – original draft, writing – review and editing, conceptualization, formal analysis. **Ailing Song**: writing – review and editing, funding acquisition, project administration, supervision. **Zirui Cui**: writing – original draft. **Manman Duanmu**: writing – original draft. **Zhipeng Ma**: writing – review and editing. **Hao Liu**: writing – review and editing. **Guangjie Shao**: writing – review and editing. **Xinyi Tan**: writing – review and editing, funding acquisition, supervision, methodology. **Guoxiu Wang**: writing – review and editing. **Hao Tian**: writing – review and editing, supervision.

## Conflicts of Interest

The authors declare no conflicts of interest.

## Data Availability

Data sharing not applicable to this article as no datasets were generated or analysed during the current study.
